# Pan-cancer analysis of the prognostic and immunological roles of SHP-1/*ptpn6*

**DOI:** 10.1038/s41598-024-74037-9

**Published:** 2024-10-04

**Authors:** Ping Cui, Jie Lian, Yang Liu, Dongsheng Zhang, Yao Lin, Lili Lu, Li Ye, Hui Chen, Sanqi An, Jiegang Huang, Hao Liang

**Affiliations:** 1 Life Science Institute, Guangxi Medical University, Nanning, China; 2 Guangxi Key Laboratory of AIDS Prevention and Treatment, Guangxi Medical University, 22, Shuangyong Road, Nanning, 530021 China; 3 School of Public Health, Guangxi Medical University, Nanning, China; 4 Geriatrics Digestion Department of Internal Medicine, The First Affiliated Hospital of Guangxi Medical University, Nanning, China; 5 Guangxi Colleges and Universities Key Laboratory of Prevention and Control of Highly Prevalent Diseases, Guangxi Medical University, Nanning, 530021 China

**Keywords:** *ptpn6*, Pan-cancer, Immune infiltration, Prognosis, Cancer prevention, Tumour immunology, Tumour immunology

## Abstract

SHP-1, a nonreceptor protein tyrosine phosphatase encoded by *ptpn6*, has been regarded as a regulatory protein of hematopoietic cell biology for years. However, there is now increasing evidence to support its role in tumors. Thus, the role of *ptpn6* for prognosis and immune regulation across 33 tumors was investigated, aiming to explore its functional heterogeneity and clinical significance in pan-cancer. Differential expression of *ptpn6* was found between cancer and adjacent normal tissues, and its expression was significantly correlated with the prognosis of tumor patients. In most cancers, *ptpn6* expression was significantly associated with immune infiltration. This was further confirmed by *ptpn6*-related genes/proteins enrichment analysis. Additionally, genetic alterations in *ptpn6* was observed in most cancers. As for epigenetic changes, it’s phosphorylation levels significantly altered in 6 tumors, while methylation levels significantly altered in 12 tumors. Notably, the methylation levels of *ptpn6* were significantly decreased in 11 tumors, accompanied by its increased expression in 8 of them, suggesting that the hypomethylation may be related to its increased expression. Our results show that *ptpn6* plays a specific role in tumor immunity and exerts a pleiotropic effect in a variety of tumors. It can serve as a prognostic factor for some cancers. Especially in LGG, KIRC, UCS and TGCT, the increased expression of *ptpn6* is associated with poor prognosis and high immune infiltration. This aids in understanding the role of *ptpn6* in tumor biology, and can provide insight into presenting a potential biomarker for poor prognosis and immune infiltration in cancers.

**Supplementary Information:**

The online version contains supplementary material available at 10.1038/s41598-024-74037-9.

## Introduction

Cancer has been a leading cause of death and a significant obstacle affecting the quality of life for decades worldwide^1^. In recent years, advances in genomic methods have expanded our knowledge about gene expression, genetic, and epigenetic alterations at the pan-genomic level in various malignancies, thus accelerating the discovery of new tumor biomarkers and therapeutic targets^2,3^.

Protein phosphatases are key regulators that control the intracellular phosphorylation required for cellular homeostasis and have received great attention in recent years as the important potential therapeutic targets for cancers and other diseases^4,5^. Among those, the SH2 domain containing protein tyrosine phosphatase (PTP), SHP-1, is a cytosolic protein tyrosine phosphatase with 595-amino acid residue encoded by *ptpn6*, which has been shown to be involved in the regulation of hematopoietic cell biology^6–9^. Recently, accumulating evidence has suggested that SHP-1 also has an impact on the occurrence and development of tumors. Its coding gene, *ptpn6*, has been reported to be overexpressed in ovarian cancer and breast cancer in previous studies^10–12^. In contrast, its expression has been shown to be diminished or abolished in T-cell lymphomas, leukemias^13–15^, hepatocellular carcinoma (HCC)^16^ and some colorectal cancers^17,18^. Furthermore, *ptpn6* has also been proven to be associated with tumor microenvironment (TME)^19–22^, which plays critical roles in tumor development involving the interaction between cancer cells, tumor infiltrating immune cells, and their supporting cells. In addition, tumor therapeutic drugs targeting SHP-1( such as several SHP-1 inhibitors) have been developed, however, results were disappointing^23^, indicating that the role of SHP-1 in tumor biology, especially in different TME, needs to be further clarified.

Taken together, increasing evidence suggests that SHP-1/*ptpn6* is possibly an immuno-raleted factor in the TME, and may play roles in regulating tumor signal transduction. Nevertheless, most studies on the role of *ptpn6* in tumors to date have been limited to a specific type of cancer. Our study carried out a pan-cancer analysis of *ptpn6* based on multiple databases to explore the heterogeneity of *ptpn6* in different cancers. We explored the expression, prognostic value, immune correlation, genetic alteration, epigenetic alteration, relevant cellular pathway and function of *ptpn6* to reveal the potential molecular pathogenesis across 33 types of cancer, which helps in comprehending the role of *ptpn6* in tumorigenesis and tumor progression from multiple perspectives.

## Materials and methods

### Differential expression analysis

The Tumor Immune Estimation Resource (TIMER) 2.0 (http://timer.cistrome.org/) is an online website used to investigate the gene expression, gene correlation and immune infiltration in tumors^24^. Difference of *ptpn6* expression between tumors and adjacent normal tissues can be obtained through the “Gene_DE” module of TIMER2. The results were validated using Gene Expression Profiling Interactive Analysis 2 (GEPIA2) database (http://gepia2.cancer-pku.cn/). The expression of *ptpn6* in different pathological stages of cancers was also obtained by GEPIA2^25^.

### Survival prognosis analysis

The heatmaps of overall survival (OS) and disease-free survival (DFS) of *ptpn6* in all TCGA tumors were acquired through GEPIA2. The corresponding survival plots with their 95% confidence interval, *p* value and hazard ratio (HR) can be obtained by the Kaplan-Meier plotter database (https://kmplot.com/analysis/)^26^. To evaluate the expression of *ptpn6* in predicting the prognosis of cancer patients, ROC analysis was conducted using the pROC package in R language (version 4.2.2).

### Immune infiltration analysis

The correlation between *ptpn6* expression and immune infiltration in pan-cancer was investigated using TIMER (https://cistrome.shinyapps.io/timer/)^27^. The tumor purity, B cells, CD8 + T cells, CD4 + T cells, macrophages, neutrophils and dendritic cells were selected. The results were visualized as scatter plots. Heatmaps and scatter plots of the correlation between *ptpn6* expression and cancer associated fibroblasts (CAFs) were generated through TIMER2^28^.

### Enrichment analysis

Enrichment analysis helps to discover novel biological functions, genotype- phenotype relationships and disease mechanisms. Experimentally determined SHP-1-binding proteins can be obtained through the STRING database (https://string-db.org/), by the following parameters: minimum required interaction score[low confidence (0.150)], meaning of network edges (evidence), maximum number of interactors to show (no more than 50 interactors in 1st shell), and active interaction sources (experiments)^29^. The top 100 *ptpn6*-related genes, were obtained by GEPIA2 and the top five genes were selected to draw the correlation scatter plot with *ptpn6*. The heat map between the selected genes and different types of tumors can be acquired through TIMER2. In addition, the intersection analysis of SHP-1-binding proteins and *ptpn6*-related genes was conducted using Jvenn (https://bioinformatics.psb.ugent.be/webtools/Venn/)^30^. These two sets of data were also combined for Kyoto Encyclopedia of Genes and Genomes (KEGG) pathway analysis^31–33^ and Gene Ontology (GO) analysis. The functional annotation data were obtained through the Database for Annotation, Visualization, and Integrated Discovery (DAVID, https://david.ncifcrf.gov/), and enriched pathways were visualized via bioinformatic (https://www.bioinformatics.com.cn/).

### Relevance of *ptpn6* across 14 functional states in distinct cancers

Single-cell RNA sequencing (scRNA-seq) can help researchers understand the functional specificity of cancer cells. CancerSEA (http://biocc.hrbmu.edu.cn/CancerSEA/) is a database for functional states of cancer cells at single-cell level, including angiogenesis, apoptosis, cell cycle, differentiation, DNA damage, DNA repair, EMT, hypoxia, inflammation, invasion, metastasis, proliferation, quiescence, and stemness^34^. The functional states of *ptpn6* in multiple cancers were explored using CancerSEA. Correlations between *ptpn6* expression and functional states in different single-cell datasets were filtered by the correlation strength > 0.3 and the *p* value < 0.05.

### Genetic alteration analysis

The cBioPortal (http://www.cbioportal.org), a comprehensive database of cancer genomics datasets^35^, is applied to the analysis of *ptpn6* genetic alteration. We explored the copy number alteration (CNA) and mutation status of *ptpn6* across all TCGA tumors using cBioPortal. The results of the alteration frequency, mutation type and CNA in various cancers were derived from the ‘Cancer Types Summary’ module. The OS, DFS, progression free survival (PFS), and disease free survival (DSS) of patients with *ptpn6* genetic altered were also obtained from cBioPortal.

### Analysis of the methylation and phosphorylation of *ptpn6*

UALCAN performed protein expression analysis from the clinical proteomic tumor analysis consortium (CPTAC) dataset and the International Cancer Proteogenome Consortium (ICPC) datasets^36^. The methylation and phosphorylation levels of *ptpn6* between different cancers and normal tissues was investigated by UALCAN database (http://ualcan.path.uab.edu/analysis.html).

### Immunohistochemistry (IHC) staining

Human Protein Atlas (HPA) (https://www.proteinatlas.org/) is a database of proteins in human organs, tissues and cells based on multiple omics approaches^37,38^. To analyze the differential expression of *ptpn6* at the protein level, the expression of *ptpn6* proteins (SHP-1) in tumor tissues and their corresponding normal tissues was downloaded from HPA and analyzed. Furthermore, the IHC images of some typical immune markers were also acquired from HPA.

### Statistical analysis

Alterations in *ptpn6* expression levels in cancer and normal tissues were estimated using two sets of t-tests. The Kaplan-Meier curve and Cox regression model were used for survival analyses in this study. The Hazard Ratio was calculated by the Cox regression model. The correlation expression analysis between the two variables was analyzed using Spearman’s or Pearson’s test. *P*-value < 0.05 was considered statistically significant.

## Results

### The expression of *ptpn6* and its correlation with cancer stage in pan-cancer

To determine differences in *ptpn6* expression between tumor and normal tissues, the *ptpn6* mRNA levels in different tumors and normal tissues were analyzed using the TIMER2 (http://timer.cistrome.org/) and GEPIA2 database (http://gepia.cancer-pku.cn/).

**Fig. 1:**
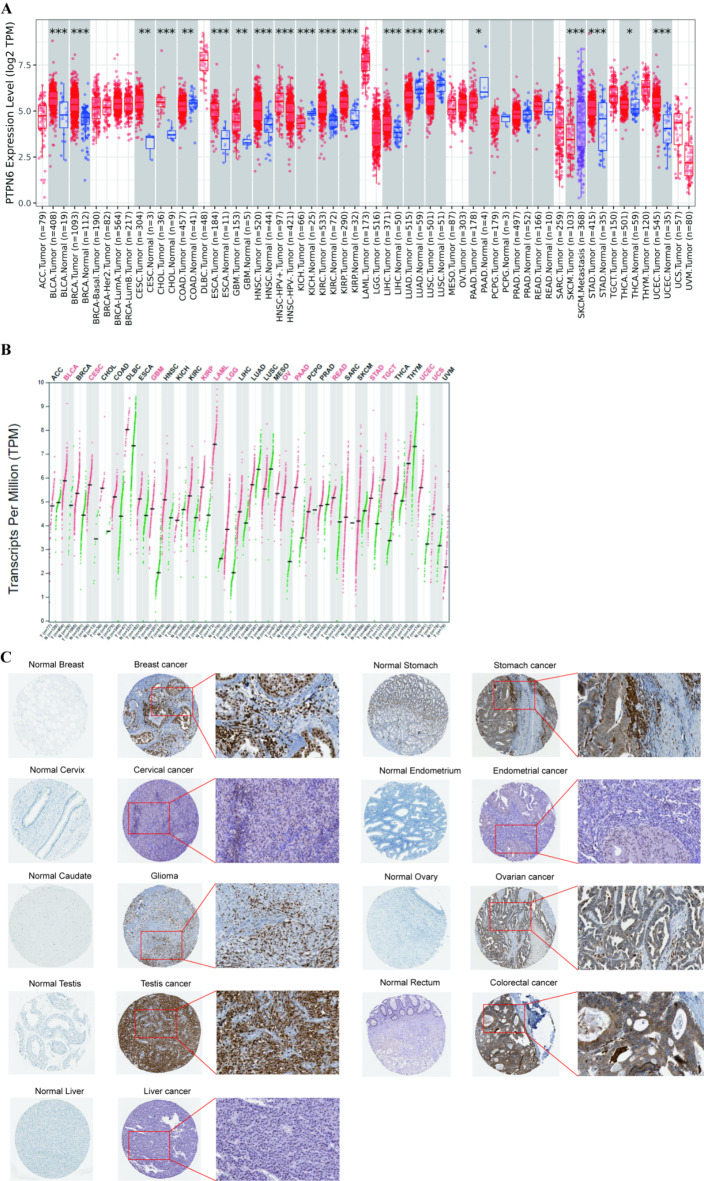
The expression levels of *ptpn6* in different types of human cancers. (**A**) Increased or decreased *ptpn6* in datasets of different cancers compared with adjacent normal tissues in TIMER2 database. (**B**) The *ptpn6* expression profile across all tumor samples and paired normal tissues from GEPIA2 database. Each dot represents the expression of the sample. The red box represents the primary tumor and the blue represents normal tissue. *p < 0. 05, **p < 0. 01, ***p < 0. 001. (**C**) The expression of *ptpn6* proteins in cancer tissues and corresponding normal tissues by HPA database.

Among the 33 types of tumors shown in TIMER2 database, the *ptpn6* expression was significantly increased in bladder urothelial carcinoma (BLCA), breast invasive carcinoma (BRCA), cervical squamous cell carcinoma and endocervical adenocarcinoma (CESC), cholangio carcinoma (CHOL), esophageal carcinoma (ESCA), glioblastoma multiforme (GBM), head and neck squamous cell carcinoma (HNSC), kidney renal clear cell carcinoma (KIRC), kidney renal papillary cell carcinoma (KIRP), liver hepatocellular carcinoma (LIHC), stomach adenocarcinoma (STAD), thyroid carcinoma (THCA), and uterine corpus endometrial carcinoma (UCEC), compared to the adjacent normal tissues (Fig. 1A). In addition, significantly lower expression was observed in colon adenocarcinoma (COAD), kidney chromophobe (KICH), lung adenocarcinoma (LUAD), pancreatic adenocarcinoma (PAAD), skin cutaneous melanoma (SKCM) and lung squamous cell carcinoma (LUSC) (Fig. 1A).

To further evaluate *ptpn6* expression in human cancers, we also examined *ptpn6* expression using the RNA-seq data from multiple malignancies in GEPIA2 database. Except for the differential expression between the tumor and adjacent normal tissues for *ptpn6* in TIMER2 database mentioned above, *ptpn6* expression was also significantly higher in acute myeloid leukemia (LAML), brain lower grade glioma (LGG), ovarian serous cystadenocarcinoma (OV), rectum adenocarcinoma (READ), testicular germ cell tumors (TGCT), and uterine carcinosarcoma (UCS) than that in paired normal tissues (Fig. 1B).

Furthermore, we performed IHC analysis of *ptpn6* expression in cancer tissues and corresponding normal tissues from the HPA website. Representative IHC images revealed that *ptpn6* was highly expressed in breast cancer, cervical cancer, glioma, testis cancer, liver cancer, stomach cancer, endometrial cancer, ovarian cancer and colorectal cancer tissues compared to their corresponding nontumorous tissue, and that its subcellular localization was in the nucleoplasm and cytoplasm (Fig. 1C).

**Fig. 2:**
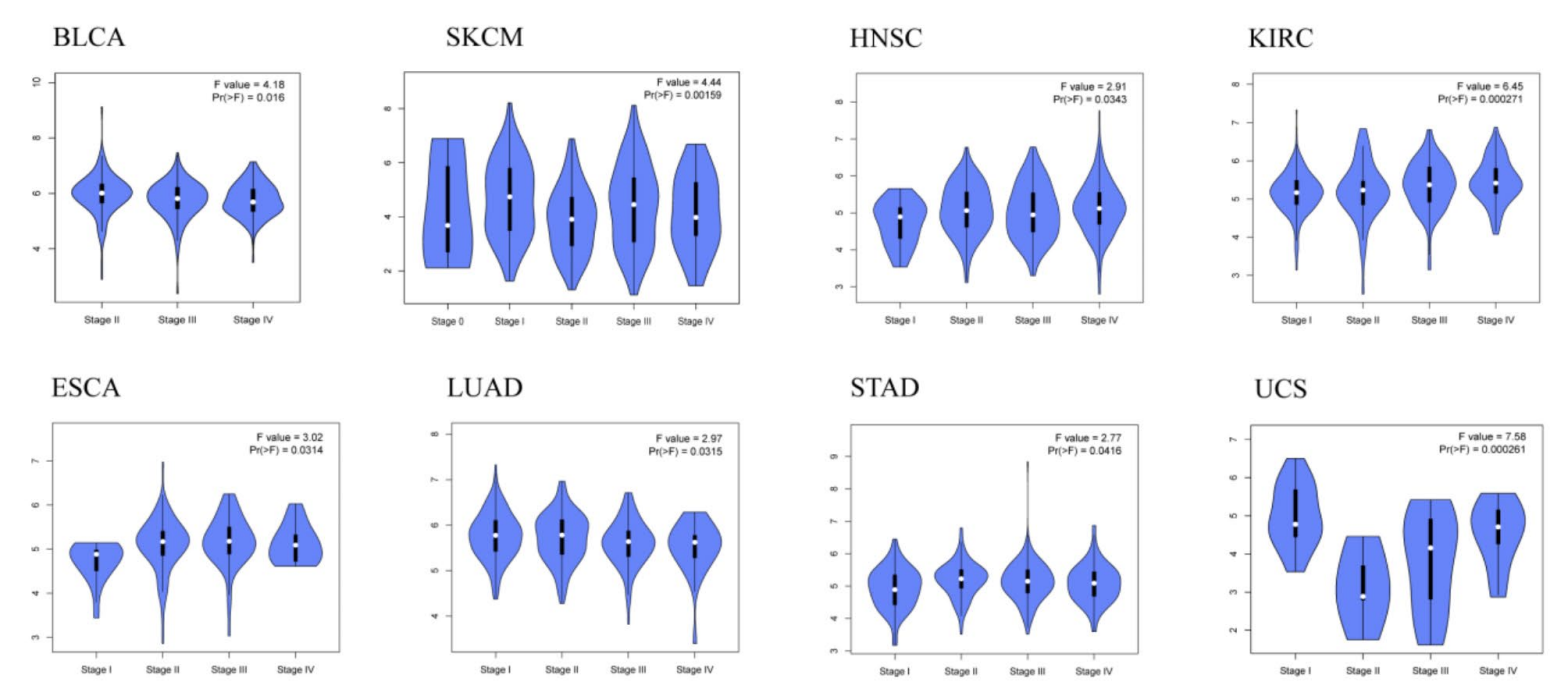
The significant correlations were observed between the *ptpn6* expression and the main pathological stages. The significance threshold was set as *p* < 0.05. The distribution of the data was given, with the white dots representing the median, and the black cylinder representing the most concentrated middle 50% of the data (i.e., 25–75% quantile). The larger the “profile” is, the more concentrated the data will be, and vice versa.

In view of the differential expression of *ptpn6* between tumor and paired normal tissues in pan-cancer, we investigated the correlations between *ptpn6* expression and cancer stage. Through the one-way ANOVA analysis of 33 tumors, we found that the expression of *ptpn6* in BLCA, ESCA, HNSC, KIRC, LUAD, SKCM, STAD and UCS was significantly positively correlated with different tumor stages (Fig. 2, *p* < 0.05). We also found that *ptpn6* expression was higher in stage III and IV compared with stage I and II in KIRC, and vice versa in LUAD, indicating that *ptpn6* might play a complex role in tumor development. However, no significant correlations were observed between *ptpn6* expression and tumor stage in the other 25 tumors.

### Prognostic potential of *ptpn6* in cancers

**Fig. 3:**
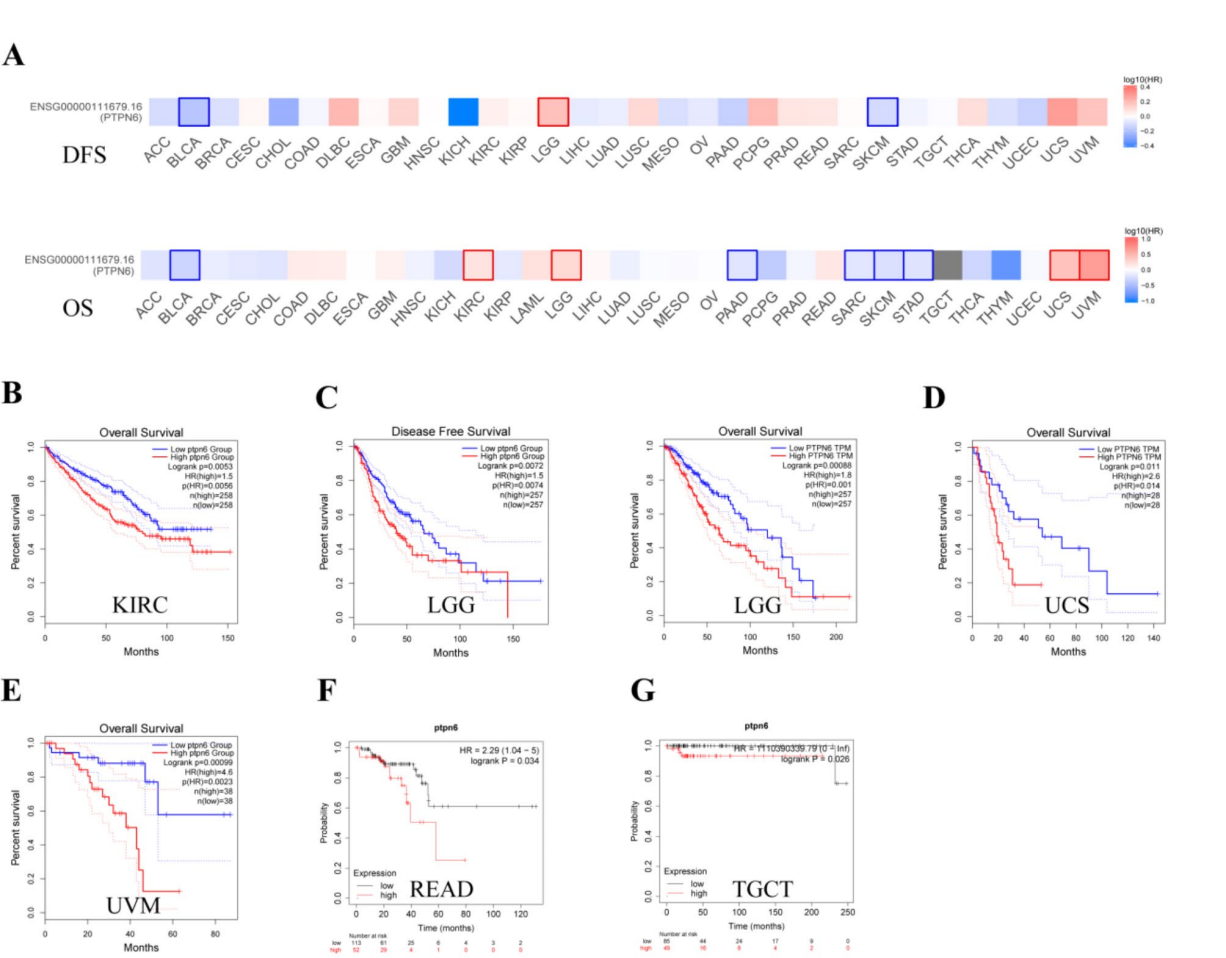
The relationship between *ptpn6* expression levels and OS/DFS in various cancer types through GEPIA2 and Kaplan-Meier. A hazard ratio (HR) greater than 1, which means the risk of death is higher in the high *ptpn6* expression cohort than in the low *ptpn6* expression cohort, is shown in red. And the reverse is shown in blue. The significant threshold was set as *p* < 0.05. (**A**) Correlation between *ptpn6* expression and survival prognosis in 33 cancer types. The survival plots with positive results were given in GEPIA2, with borders indicating statistically significant results. The darker the color is, the further away the HR is from 1. (**B**–**G**) The correlation between high *ptpn6* expression and prognosis of different tumor types were explored by GEPIA2 (**B**–**E**) and Kaplan-Meier (**F**,**G**) database.

To explore whether *ptpn6* expression was related to the survival indicators of cancer patients, overall survival (OS) and disease free survival (DFS) analyses were performed. The cancer patients were divided into high-*ptpn6-*expression and low-*ptpn6*-expression groups, and the association of *ptpn6* expression with the patient prognosis in multiple cancers was explored via Kaplan-Meier plotter database and GEPIA2 database. Cox regression analysis of the results from 33 types of cancer suggested that the *ptpn6* expression significantly correlated with OS in 13 types of cancer, including BLCA, BRCA, CESC, KIRC, KIRP, LUAD, PAAD, READ, SARC, STAD, TGCT, THYM and UCEC ( Figure S1, all *P* < 0.05 ). The results from GEPIA2 datasets showed that high *ptpn6* expression levels were associated with poor prognosis in KIRC (*p* = 0.0056), LGG (*p* = 0.001), UCS (*p* = 0.014) and UVM (*p* = 0.0023) (Fig. 3A-E). We also analyzed the prognostic values of *ptpn6* in pan-cancer using Kaplan-Meier database, and found that high expression of *ptpn6* were significantly correlated with the shortened OS of TGCT (*p* = 0.026) and READ patients (*p* = 0.034) (Fig. 3F-G). These results suggested that the *ptpn6* expression had an impact on the prognosis in KIRC, LGG, UCS, UVM, TGCT and READ. Therefore, it is conceivable that high *ptpn6* expression is an independent risk factor leading to poor prognosis of these 6 types, which we will focus on in the following studies.

Meanwhile, high *ptpn6* expression was correlated with good prognosis in BLCA, PAAD, sarcoma (SARC), SKCM, STAD, BRCA, CESC, KIRP, LUAD, thymoma (THYM) and UCEC from GEPIA2 and Kaplan-Meier plotter database (Fig. 3A, S1-2, all *P* < 0.05), suggesting the complexity of the correlation between *ptpn6* expression and prognosis of different tumor types.

### *Ptpn6* expression is correlated with immune infiltration in most types of cancer

A growing body of evidence suggests that tumor progression and prognosis are closely related to the TME. During tumor progression, a variety of immune cells are recruited into the microenvironment surrounding the tumor cells, infiltrate into the tumor and become tumor immune infiltration^20,39,40^. In addition, tumor purity is an important factor influencing the analysis of immune infiltration in clinical tumor samples by genomic approaches^41^, and low tumor purity is an independent risk factor for poor prognosis in some cancer types^42,43^. To investigate the relationship between *ptpn6* expression and tumor immune infiltration, we assessed the correlations between *ptpn6* expression and immune infiltration levels in 32 cancer types from TIMER. The results showed that *ptpn6* expression was significantly correlated with tumor purity in 21 types of cancer (Table S1). In addition, *ptpn6* expression was significantly correlated with infiltrating levels of B cell in 27 types of cancers, CD8 + T cells in 19 types of cancer, CD4 + T cells in 26 types of cancer, macrophages in 21 types of cancer, neutrophils in 23 types of cancer, and dendritic cells in 29 types of cancer (Table S1).

**Fig. 4:**
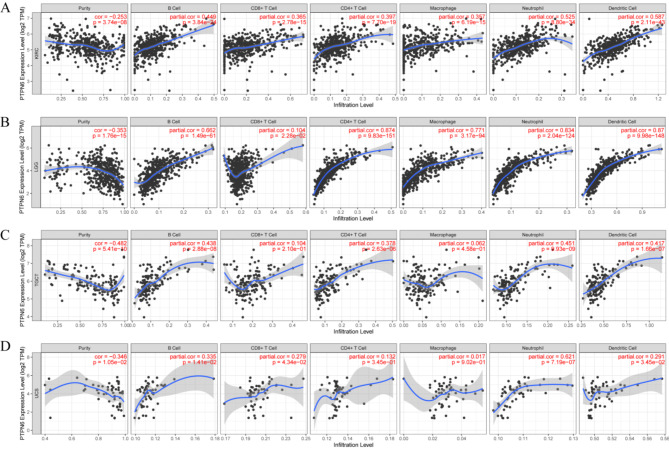
Correlation of *ptpn6* expression with immune infiltration levels in KIRC, LGG, TGCT, and UCS. (**A**,**B**) *Ptpn6* expression was significantly negatively correlated with tumor purity and positively correlated with infiltrating levels of B cells, CD8 + T cells, CD4 + T cells, macrophages, neutrophils, and dendritic cells in KIRC and LGG. (**C**) *Ptpn6* expression had significant negative correlations with tumor purity and positive correlations with infiltrating levels of B cells, CD4 + T cells, macrophages, neutrophils, and dendritic cells in TGCT, except for CD8 + T cells and macrophages. (**D**) *Ptpn6* expression was significantly negatively correlated with tumor purity, and positively correlated with infiltrating levels of B cells, CD8 + T cells, macrophages, neutrophils, and dendritic cells in UCS, but not significantly correlated with infiltrating level of CD4 + T cells and macrophages.

Notably, among the 6 cancers previously screened for which high *ptpn6* expression was associated with poor prognosis, *ptpn6* expression was significantly negatively related to tumor purity in 4 of them, including KIRC, LGG, TGCT and UCS (Fig. 4). In addition, *ptpn6* expression had significant positive correlations with infiltrating levels of B cells, CD8 + T cells, CD4 + T cells, macrophages, neutrophils, and dendritic cells in KIRC and LGG (Fig. 4A-B). In terms of TGCT and UCS, it also had significant positive correlations with infiltrating levels of most types of cells, but no significant correlation with infiltrating level of CD8 + T cells and macrophages in TGCT, CD4 + T cells and macrophages in UCS (Fig. 4C-D). *Ptpn6* expression was positively correlated with infiltrating levels of CD4 + T cells and Dendritic cells in READ, B cells in UVM (Table S1). These results showed that *ptpn6* expression was correlated with immune infiltration in most types of cancer, and its high expression was correlated with poor prognosis and high immune infiltration in KIRC, LGG, TGCT, and UCS. All these revealed the specific role of *ptpn6* in regulating immune infiltration and its aberrant expression may alter TME, directly or indirectly, thereby affecting tumor prognosis.

### Enrichment analysis of *ptpn6*-related genes/proteins

**Fig. 5:**
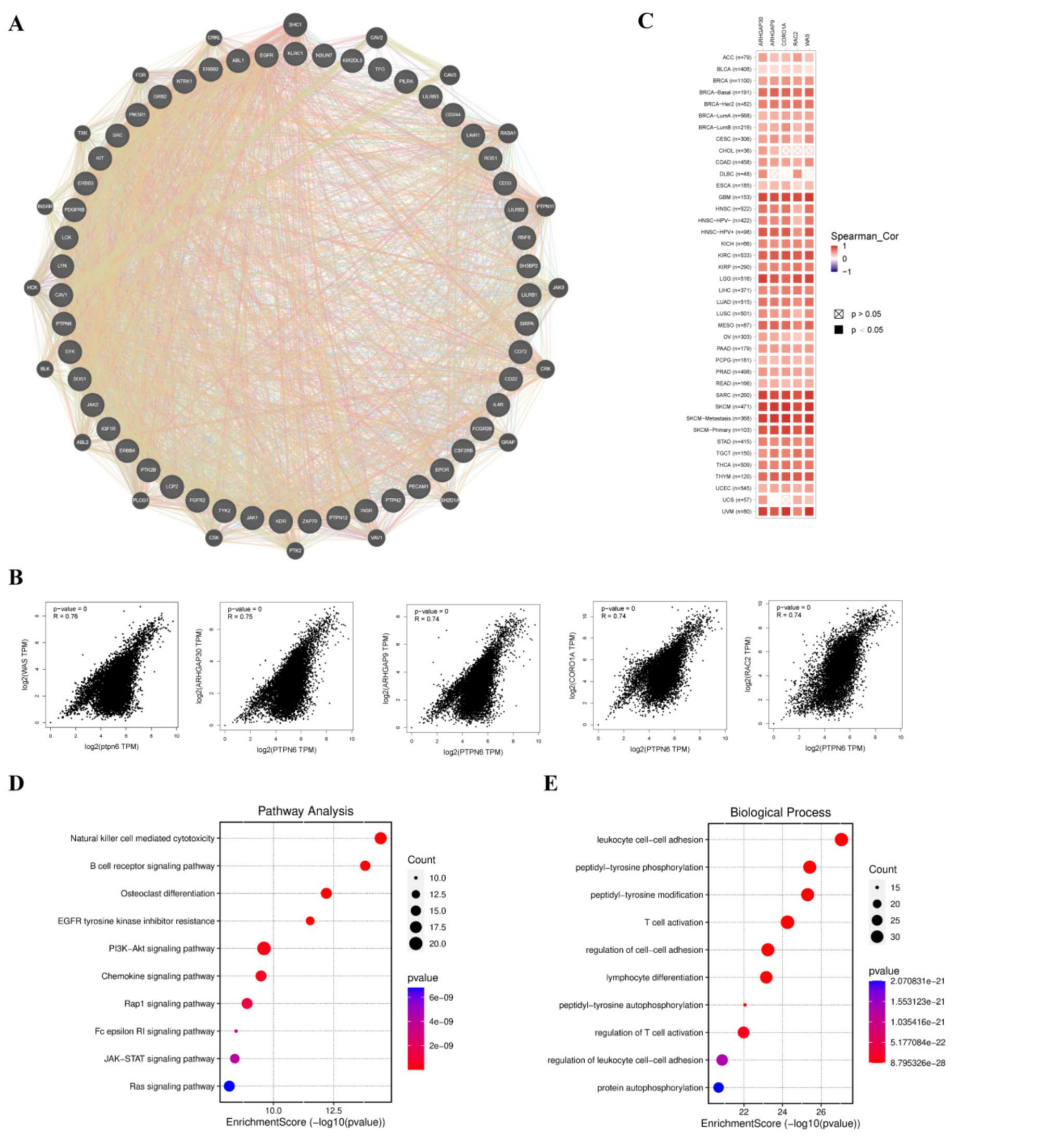
The *ptpn6*-related genes/proteins enrichment analysis. (**A**) The available experimentally determined SHP-1-binding proteins from the STRING database and their interaction network. (**B**) The correlation of the expression between *ptpn6* and the top five *ptpn6*-related genes, including WAS, ARHGAP30, ARHGAP9, CORO1A and RAC2 (*R* = 0.76, 0.75, 0.74, 0.74, 0.74). (**C**) The corresponding heatmap between these five genes and *ptpn6* in the 33 types of cancer. The significant threshold was set as *p* < 0.05. Red indicates a positive correlation, while blue means the opposite. The darker the color is, the closer the absolute value of the correlation coefficient is to 1, and the higher the correlation is. (**D**,**E**) KEGG pathway analysis and GO enrichment analysis for the SHP-1-binding proteins and *ptpn6*-related genes.

The enrichment analysis of *ptpn6*-related genes and SHP-1 binding proteins was conducted to study the biological functions of *ptpn6* in tumors, so as to further reveal its molecular mechanism in tumorigenesis and tumor progression. A batch of SHP-1-binding proteins supported by experimental evidence and *ptpn6* expression-related genes were obtained by the STRING and GEPIA2 databases, respectively, from which the top 50 SHP-1-binding proteins (Fig. 5A) and the top 100 highest positively correlated genes with *ptpn6* (hereinafter named as *ptpn6*-related genes) were selected for further study as described in previous studies^44^.

The interaction network of 50 SHP-1-binding proteins showed that many of them were involved in immune function and cancer signal transduction, such as IL4R, JAK1, CD33 (Fig. 5A). And most *ptpn6*-related genes were related to tumorigenesis, infiltration and immune regulation, including ARHGAP30, ARHGAP9, CORO1A, RAC2, FERMT3, VAV1, RASAL3, etc. According to the correlation coefficient (R), the top 5 genes were WAS (*R* = 0.76), ARHGAP30 (*R* = 0.75), ARHGAP9 (*R* = 0.74), CORO1A (*R* = 0.74) and RAC2 (*R* = 0.74) (Fig. 5B). Furthermore, the correlation scatter plots and corresponding heatmaps were generated between the five genes and *ptpn6* in 33 cancers, respectively, and it indicated that *ptpn6* was positively correlated with these five genes in the majority of cancer types (Fig. 5B-C). In addition, an intersection analysis of SHP-1-binding proteins and *ptpn6*-related genes was also conducted and two common members LCP2 and LAIR1 were found.

To further investigate the molecular mechanisms of *ptpn6* in tumors, the *ptpn6*-related genes and SHP-1-binding proteins were combined for KEGG analysis and GO analysis. The KEGG results suggested that *ptpn6* was closely related to several important cancer and immune related processes, such as JAK-STAT signaling pathway, PI3K-AKT signaling pathway, natural killer cell mediated cytotoxicity, B cell receptor signaling pathway, and PD-L1 expression and PD-1 checkpoint pathway in cancer (Fig. 5D). GO enrichment analysis revealed that many of these genes were enriched in peptidyl-tyrosine modification, protein autophosphorylation, lymphocyte differentiation, T cell activation and regulation of T cell activation (Fig. 5E).

All these results showed that most of *ptpn6*-related genes/proteins were involved in the occurrence and development of tumors, as well as the immunity, suggesting the important role of *ptpn6* in tumors and their immunity.

### Functional states of *ptpn6* across different cancer types

The high functional heterogeneity of cancer cells plays a crucial role in cancer research, which is of great significance to comprehensively explore the different functional states of cancer cells at the single-cell level^34^. The functional states of *ptpn6* were analyzed at single-cell resolution in 14 cancers, including LUAD, non-small cell lung cancer (NSCLC), renal cell carcinoma (RCC), acute lymphoblastic leukemia (ALL), prostate cancer (PC), retinoblastoma (RB), GBM, uveal melanoma (UVM/UM), colorectal cancer (CRC), OV, etc.

**Fig. 6:**
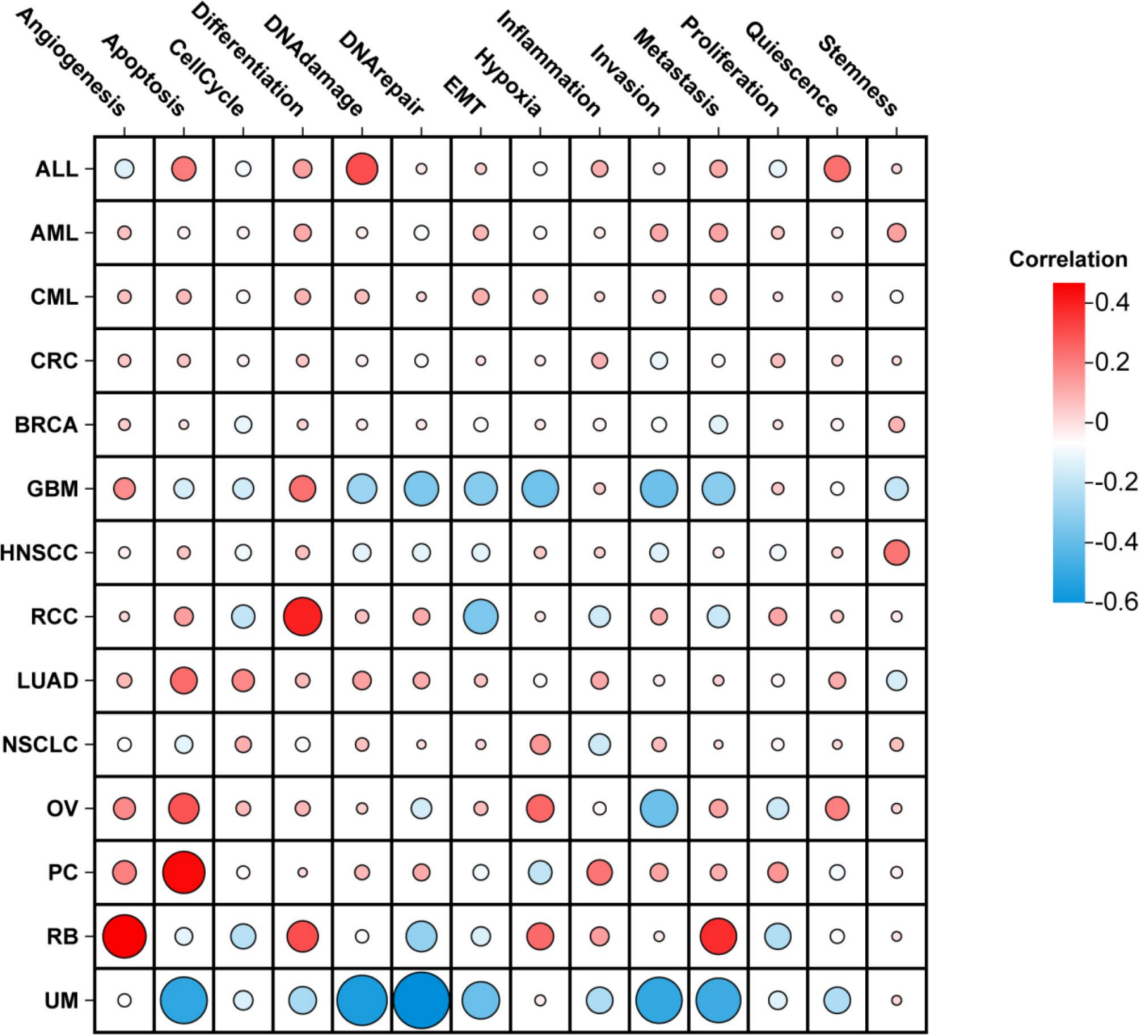
Average correlations between *ptpn6 *and functional states in different cancers. The bar chart shows the number of datasets in which the gene *ptpn6* was significantly related to the corresponding state. Correlations between *ptpn6* expression and functional states in different single-cell datasets were screened using correlation strength (cor) > 0.3 and the *p*-value < 0.05. The different colors in the figure represent the positive (red) or negative (blue) correlation of *ptpn6* expression and functional states, and the size of the dots represents correlation strength (cor).

We observed that *ptpn6* was correlated with multiple functional states in multiple cancers. In UVM/UM, 6 functional states were negatively correlated with the gene *ptpn6*, including Apoptosis (cor = -0.515), DNA damage (cor = -0.562), DNA repair (cor = -0.647), EMT (cor =-0.389), Invasion (cor = -0.507), and Metastasis (cor = -0.487) (Fig. 6). In GBM, there were 5 functional states, DNA repair (cor = -0.346), EMT (cor =-0.327), Hypoxia (cor =-0.376), Invasion (cor = -0.384), and Metastasis (cor = -0.323), negatively correlated with *ptpn6* (Fig. 6). In addition, *ptpn6* was negatively with Invasion in OV (cor = -0.387) (Fig. 6). On the other hand, the expression of *ptpn6* was positively correlated with Angiogenesis (cor = 0.468), Differentiation (cor = 0.305) and Metastasis (cor = 0.373) in RB, DNA damage in ALL (cor = 0.305), Differentiation in RCC (cor = 0.398), and Apoptosis in PC (cor = 0.452) (Fig. 6). These results suggested that *ptpn6* might play an important role by regulating multiple functions in pan-cancer.

### The genetic alterations of *ptpn6* in pan-cancer

**Fig. 7:**
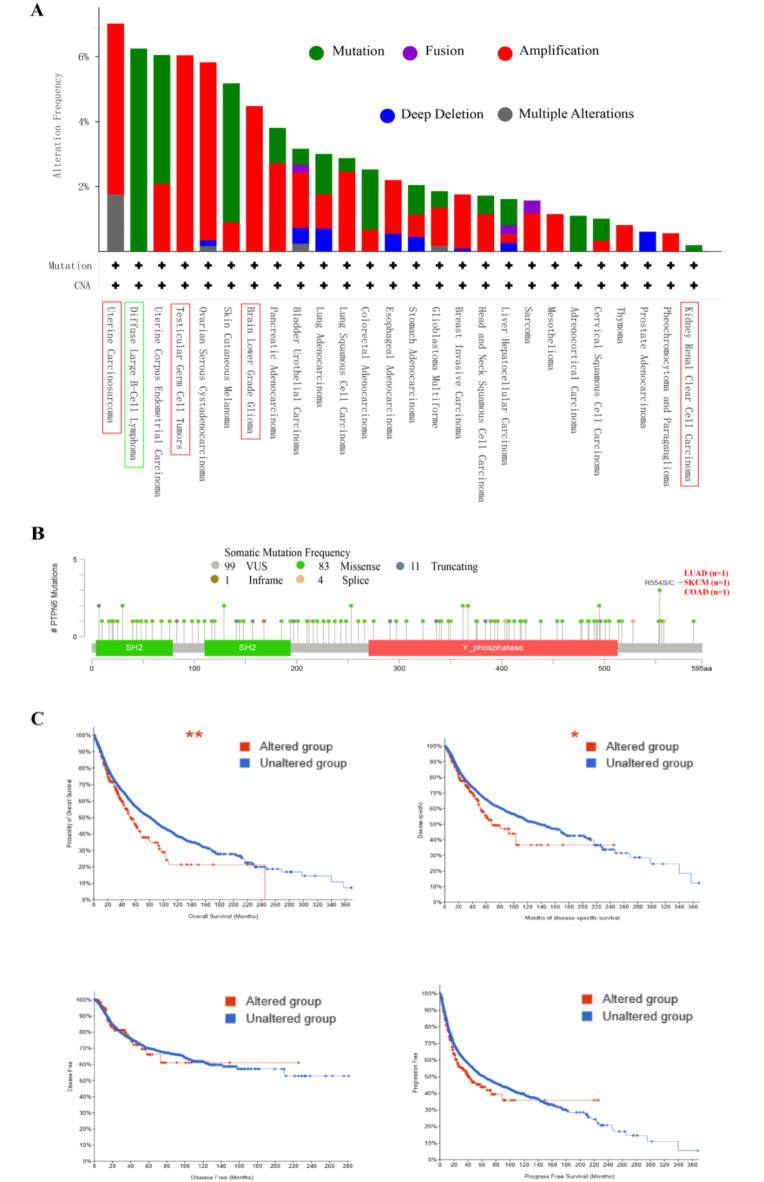
The genetic alteration of *ptpn6 *in different tumors using the cBioPortal database. (**A**) The alteration frequency and type of *ptpn6* in 32 TCGA tumor types. (**B**) The map of types, sites and case numbers of *ptpn6* mutation. (**C**) The effect of *ptpn6* genetic alteration on OS, DSS, DFS and PFS. *p < 0. 05, **p < 0. 01, ***p < 0. 001.

In order to further investigate the molecular mechanism of *ptpn6* in tumorigenesis, we used cBioPortal (https://www.cbioportal. org) to evaluate the genetic alteration of *ptpn6* in different tumor samples of the TCGA cohorts. The alteration frequency, mutation type and copy number change (CNA) of *ptpn6* were analyzed in 10,967 samples of 32 TCGA tumors. Genetic change of *ptpn6* was found in 26 tumors, including “mutation”, “amplification”, “deep deletion”, “multiple alterations” and “fusion” (Fig. 7A). Notably, the majority of the investigated cancers exhibited either “amplification” or “mutation” as the predominant form of genetic alteration (Fig. 7A).

Among the 4 types of cancers selected previously, *ptpn6* alteration frequency was relatively higher in UCS, TGCT and LGG, and the highest alteration frequency was observed in UCS patients, mainly characterized by “amplification” and “multiple alterations” (Fig. 7A). Similarly, the alteration of *ptpn6* gene was mainly manifested as “amplification” in TGCT and LGG patients, and “mutation” in KIRC patients (Fig. 7A). Besides the tumor types described above, diffuse large B cell lymphoma (DLBC) was also found with higher frequency of *ptpn6* alterations, with “mutation” as the only alteration type (Fig. 7A).

Subsequently, we investigated the sites and types of *ptpn6 “*mutation” through cBioPortal Mutations Module, and found that among the 99 “mutation” samples included in this database, the first ranked mutation type was missense mutation, with 83 recorded samples, among which, R554S/C missense mutation was the most frequent (Fig. 7B).

We further investigated the relationship between *ptpn6* alteration and prognosis in pan-cancer, and found that tumor patients with genetic alterations in *ptpn6* had worse OS *(p* = 8.571e-3) and DSS *(p* = 0.0412) than patients without alterations, but no difference in DFS *(p* = 0.994) and PFS *(p* = 0.0804) between the two groups (Fig. 7C). These findings suggested that the genetic alteration of *ptpn6* might lead to poor clinical prognosis.

### The *ptpn6* phosphorylation heterogeneity in pan-cancer

Posttranslational modification (PTM) is a key molecular mechanism of *ptpn6* activation^45^, and phosphorylation is an important one. In previous studies, *ptpn6* has been shown to possess the ability to phosphorylate the receptor through its SH2 domain, which is also a vital region for phosphorylation of itself^46^. In order to investigate the relationship between the alterations in *ptpn6* phosphorylation and cancer progression, the *ptpn6* phosphorylation were analyzed in tumor and normal tissues through UALCAN dataset.

**Fig. 8:**
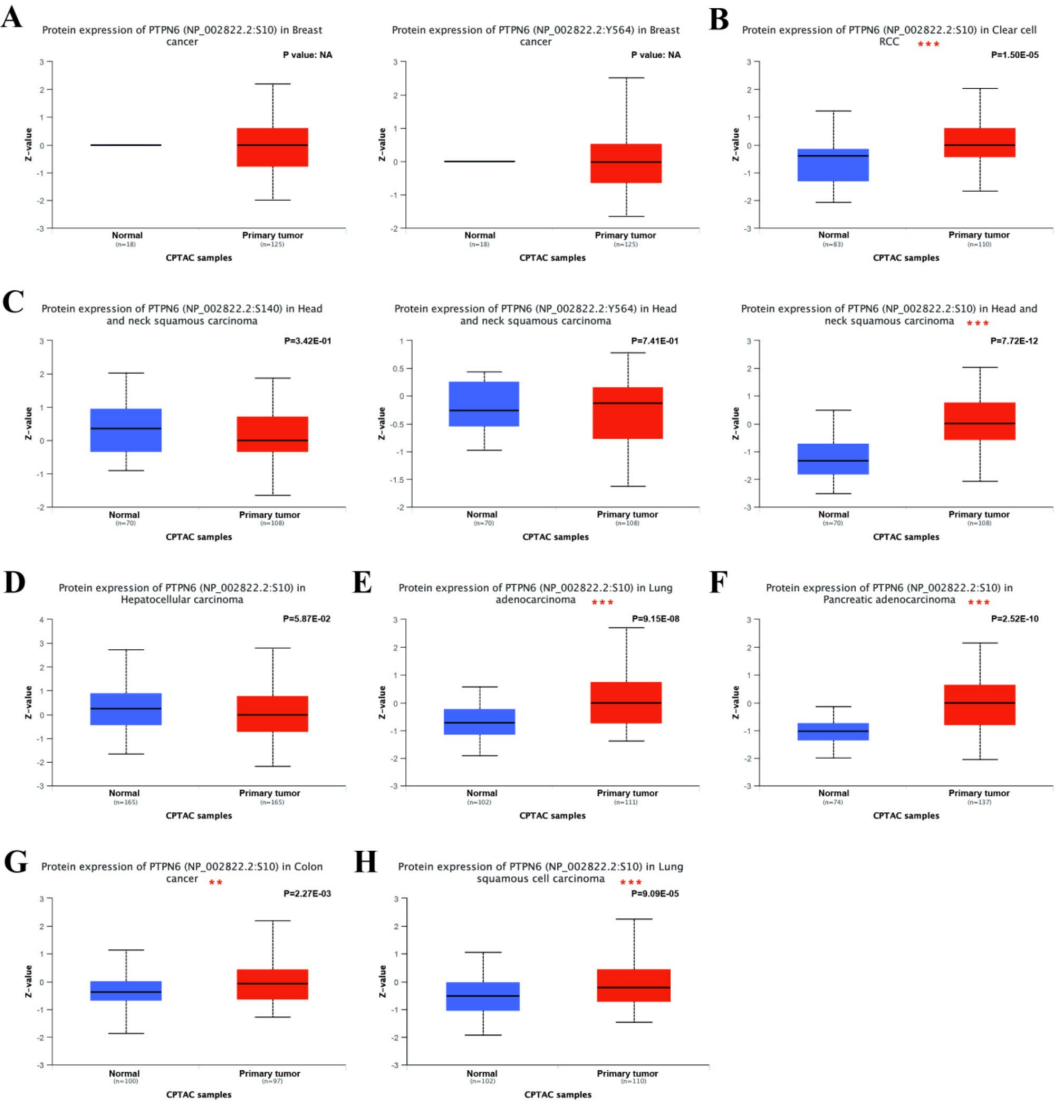
Phosphorylation analysis of *ptpn6 *in different tumors from the UALCAN database. The *ptpn6* phosphorylation was analyzed between primary tissues of selected tumor and normal tissues, including breast cancer (**A**), clear cell RCC (**B**), HNSC (**C**), hepatocellular carcinoma (**D**), LUAD (**E**), PAAD (**F**), colon cancer (**G**) and LUSC (**H**). Z-values represent standard deviations from the median across samples for the given cancer type. The log2 Spectral count ratio values from CPTAC were first normalized within each sample profile, then normalized across samples. The red box indicates primary tumors, and the blue shows normal tissues. *p < 0. 05, **p < 0. 01, ***p < 0. 001.

The phosphorylation from UALCAN dataset indicated that significantly altered *ptpn6* phosphorylation levels were observed in 6 tumors, including clear cell RCC (CCRCC), LUAD, HNSC, PAAD, colon cancer (CC) and LUSC, and the changes were mainly distributed in three sites, including S10, Y564 and S140 (Fig. 8). Compared with normal tissues, the phosphorylation level of S10 site of *ptpn6* was significantly increased in CCRCC, LUAD, HNSC, CC, LUSC and PAAD tumor tissues (Fig. 8, all *p* < 0.05). In HNSC, the phosphorylation level of *ptpn6* increased at Y564 and decreased at S140, but the difference was not significant (Fig. 8).

### The promoter methylation heterogeneity of *ptpn6* exists in different tumors

DNA methylation is one of the most intensely studied epigenetic modifications affecting occurrence and progression of cancer, and changes in DNA methylation in cancer have been considered as important biomarkers for diagnosis and prognosis^45,47^. By UALCAN databases, we investigated the promoter methylation of *ptpn6* in 32 kinds of tumors, thus to analyze the methylation heterogeneity of *ptpn6* in cancer.

**Fig. 9:**
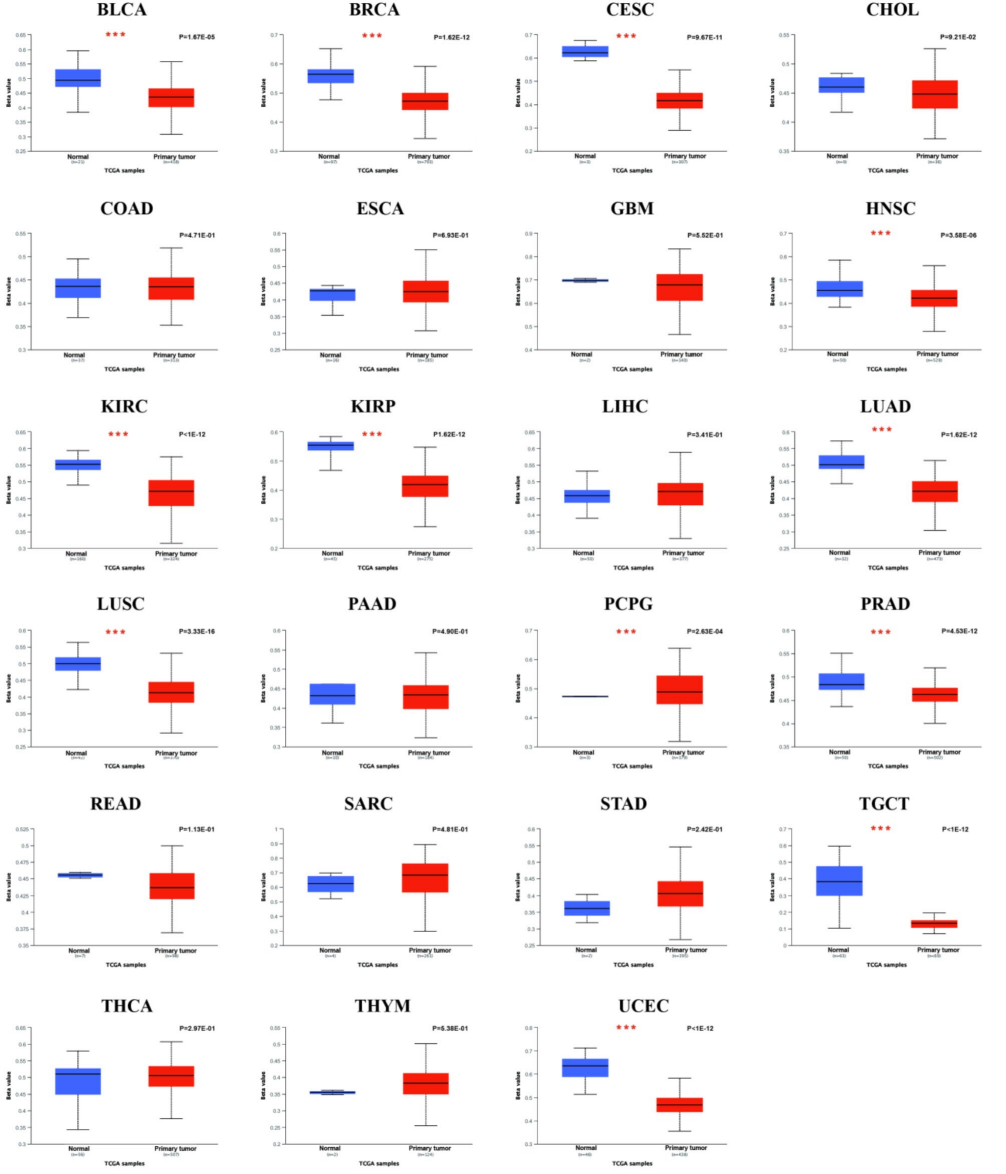
The *ptpn6 *promoter methylation levels in different cancers were obtained from UALCAN. The Beta value indicates level of promotor methylation, ranging from 0 (unmethylated) to 1 (fully methylated). Different beta value cut-off has been considered to indicate hyper-methylation [Beta value: 0.7 − 0.5] or hypo-methylation [Beta-value: 0.3 − 0.25]. The red box indicates primary tumors, while blue box indicates normal tissues. The significant threshold was set as *p* < 0.05. *p < 0. 05, **p < 0. 01, ***p < 0. 001.

We found that the promoter methylation of *ptpn6* was significantly different in 12 tumors compared to the corresponding normal tissues. Among them, the significant decrease in the promoter methylation levels of *ptpn6* was observed in tissues of 11 tumors, including BLCA, BRCA, CESC, HNSC, KIRC, KIRP, LUAD, LUSC, prostate adenocarcinoma (PRAD), TGCT and UCEC, compared to normal tissues (Fig. 9). As described before, the expression of *ptpn6* was significantly increased in 8 of these tumors (BLCA, BRCA, CESC, HNSC, KIRC, KIRP, TGCT and UCEC), than that in normal tissues. It was indicated that hypomethylation of *ptpn6* may be related to its increased expression. Among those, as previously mentioned, the increased expression of *ptpn6* in KIRC and TGCT is significantly correlated with poor prognosis and high immune infiltration, reflecting the potential associations of *ptpn6* promoter methylation, gene expression, and its role in cancer prognosis and immune infiltration.

In addition, in pheochromocytoma and paraganglioma (PCPG), the methylation level of *ptpn6* was significantly increased (Fig. 9). No significant differences were observed between COAD, ESCA, CHOL, GBM, LIHC, PAAD, READ, SARC, STAD, THCA and THYM tissues and matched normal tissues (Fig. 9).

## Discussion

*Ptpn6* is a signaling molecule that regulates various cellular processes, including cell growth, differentiation, mitotic cycle and oncogenic transformation, and plays an important role in the occurrence and development of cancer^16,48–51^. In order to better investigate the heterogeneity of *ptpn6* in tumors, we conducted a systematic pan-cancer analysis. Our results offer insight into the prognostic and immunological roles of *ptpn6* across different tumors, indicating that *ptpn6* exerts a pleiotropic effect on malignancy and it may play roles in many aspects of tumor biology.

Herein, we analyzed the difference in *ptpn6* expression in 33 tumors and the normal tissues, and found that *ptpn6* expression was significantly different in 25 tumors. Among them, the expression of *ptpn6* was significantly higher in 20 tumors, and its high expression was associated with poor prognostic in 6 of them, including KIRC, LGG, UCS, UVM, TGCT and READ, which indicated that *ptpn6* may have a cancer-promoting effect in some cancer types. This is consistent with the reports on cancer-promoting effects of *ptpn6* in LGG and KIRC^52,53^. In addition, receiver-operating characteristic (ROC) curves were used to explore the expression of *ptpn6* and the prediction of survival prognosis in 5 of those tumors, and it showed that *ptpn6* expression had some predictive value for KIRC (AUC = 0.619), LGG (AUC = 0.572), TGCT (AUC = 0.606), and UVM (AUC = 0.617) (Figure S3). These results confirmed the prognostic role of *ptpn6*, which may be a valuable prognostic biomarker for some specific types of cancer.

TME features serve as markers for evaluating tumor cell responses to immunotherapy and influence clinical outcomes^54^. More and more studies have suggested that tumor immune infiltrating cells, including but not limited to B cells, T cells, neutrophils, macrophages, dendritic cell (DC), and tumor associated fibroblast (CAF), are closely associated with the tumor progression. Thus, the associations between *ptpn6* expression and immune infiltration levels across 33 types of cancer was explored, and the results showed that *ptpn6* expression was significantly associated with tumor immune infiltration in most cancers. Especially in LGG, KIRC, UCS and TGCT, the increased expression of *ptpn6* was significantly associated with poor prognosis and high immune infiltration. We further carried out IHC analyses of typical immune markers in kidney cancer, testicular cancer, brain glioma and uterine carcinoma through HPA database, and it revealed that compared with the corresponding non-tumor tissues, the immune markers HIF1A, IDH1, AFP and VIM were highly expressed in these four cancer tissues, respectively (Figure S5A). And *ptpn6* expression was significantly correlated with the markers HIF1A and IDH1(Figure S5B). All these suggested that *ptpn6* plays a specific role in tumor immunity, such as a key regulatory role in the interaction between cancer cells and various components of TME, thereby affect immune escape of tumors and ultimately tumor proliferation, recurrence, or metastasis. In addition, we also investigated the correlation between CAFs and *ptpn6* expression, and found that *ptpn6* expression was positively correlated with CAFs in COAD, HNSC-HPV-, LGG and THCA, while negatively correlated with BRCA-LumA/LumB, mesothelioma (MESO) and THYM (Figure S4).

Subsequent studies on *ptpn6-*related genes and proteins obtained a large number of immune-related and tumor-related proteins, further confirming the role of *ptpn6* in immunity and tumorigenesis. For example, CORO1A inhibited TLR-mediated signal activation in human macrophages^55^, RAC2 mutations led to different forms of primary immunodeficiencies^56^, RASAL3 served as a therapeutic target to regulate inflammatory responses in many inflammatory disease states^57^, ARHGAP30 and ARHGAP9 genetic alterations led to carcinogenesis of colorectal cancer (CRC)^58^, Vav1 accelerated Ras-driven lung cancer and modulates its tumor microenvironment^59,60^, and FERMT3 acted as a tumor suppressor gene in GBM and lung cancer^61,62^. Furthermore, our researches have found two important intersection proteins, LCP2 and LAIR1, which possibly had potential prognostic value or serve as immunotherapeutic targets for cancers. LCP2 had been confirmed to be an independent prognostic factor in DLBCL, LUAD and melanoma^39,63,64^. Previous studies have also demonstrated that LAIR1 induced T cell exhaustion through regulating the expression of *ptpn6*, leading to the occurrence and development of lung tumors^19^.

In addition, through the KEGG and GO enrichment analysis of the *ptpn6*-related genes and SHP-1-binding proteins, we could find that *ptpn6* is involved in immune-related biological processes, such as natural killer cell mediated cytotoxicity, B cell receptor signaling pathway, T cell receptor signaling pathway, further indicating its critical role in immune function. However, it is still unclear about the correlation between *ptpn6* and these genes/proteins. Therefore, further study of other *ptpn6* expression-correlated genes/proteins, such as whether *ptpn6* can regulate the expression of these genes, and affect the immune infiltration, thus affecting the occurrence and development of tumors, are of great significance.

In addition, genomic DNA methylation is an important epigenetic event in humans, and the alterations of methylation patterns may play important roles in tumorigenesis^24,65,66^. As one of the earliest molecular changes during the transformation process of normal cells into cancerous cells^67^, aberrant DNA methylation pattern may have potential applications in the early detection of malignancies. Currently, many studies have been devoted to evaluating epigenetic regulation of gene expression by promoter methylation, with the aim of identifying new biomarkers of cancer development. In our results from the database, the methylation levels of *ptpn6* were significantly decreased in 11 types of tumors, along with the significantly higher expression of *ptpn6* in 8 types of which, including BLCA, BRCA, CESC, HNSC, KIRC, KIRP, TGCT and UCEC, than that in normal tissues. In previous studies, the hypermethylation of the *ptpn6* promoter was observed in leukemia and lymphoma^14,68–70^, esophageal squamous cell carcinoma^71^, gastric adenocarcinoma^72^, breast cancer^73^, endometrial carcinoma^54^, and nasopharyngeal carcinoma^74^. Moreover, the expression level of *ptpn6* has a strong reverse correlation with promoter methylation, and the promoter hypermethylation has been proved to be the cause of *ptpn6* silencing^47^. Here, the hypomethylation of *ptpn6* was associated with increased expression of *ptpn6.* Notably, in KIRC and TGCT, the increased expression of *ptpn6* was associated with its poor prognosis and high immunoinfiltration, accompanied by the promoter hypomethylation, reflecting the close relationship between epigenetic modifications (such as methylation) and gene expression, cancer immune environment and prognosis. Thus, we speculate that promoter hypomethylation of *ptpn6* in some cancers may lead to its increased expression, which may be associated with the low prognosis and high immunoinfiltration, according to our results. Nevertheless, it is worth noting that the regulation of *ptpn6* promoter methylation is complex and involved in multiple factors, such as environmental exposure, gene mutations, and epigenetic modifications. Therefore, it is necessary to conduct comprehensive and systematic research on the mechanisms of *ptpn6* promoter methylation in cancer, in order to provide a scientific basis for its clinical application. Our findings can provide clues for further work related to *ptpn6* methylation, which holds great promise for revealing the mechanism of *ptpn6* in tumorigenesis and progression.

Our findings suggest that *ptpn6* can be served as an independent prognostic factor of many tumors and for different tumors, its expression level will bring different or even completely opposite prognostic outcomes. In this study, the increased expression of *ptpn6* was found to be significantly associated with poor prognosis and high immune infiltration in LGG, KIRC, UCS, and TGCT, suggesting its cancer-promoting role in some specific tumor types, as we have described previously. However, in BLCA, BRCA, CESC, STAD, KIRP, PAAD, and UCEC, its high expression was significantly associated with good prognosis(Figure S1-2). In previous functional verification experiments, it was confirmed that *ptpn6* can restrict the growth of PAAD cells by reducing the phosphorylation of STAT3 and blocking the activation of STAT3^75^. It was also found that increased *ptpn6* levels blocked the JAK2/STAT3 pathway, leading to reduced proliferation, migration, and invasion of STAD^72,76,77^. These results further validated our findings regarding the role of *ptpn6* in STAD and PAAD. These suggest a complex correlation between *ptpn6* expression and the survival of different types of cancer, possibly due to its different biological backgrounds and roles in tumorigenesis. This might, at least in part, contribute to the disappointing results of drug discovery to date with SHP-1 as a tumor therapeutic target. The in-depth study of the specific role of *ptpn6* in each cancer may have important implications for diagnosis and immunotherapy in the era of precision medicine.

## Conclusions

In this study, we provide a comprehensive understanding of the roles of *ptpn6* across different tumors, including the expression, prognosis, immune correlation, genetic alteration, epigenetic alterations, and relevant cellular pathways and functions. The results showed that *ptpn6* can serve as a prognostic factor for a variety of cancers, and that *ptpn6* can play an important role in tumor immunity by affecting tumor infiltrating immune cells. These findings aid in understanding the role of *ptpn6* in tumorigenesis and development, presenting a potential biomarker for poor prognosis and immune infiltration in diverse cancer types.

## Summary

SHP-1, a nonreceptor protein tyrosine phosphatase encoded by *ptpn6*, has been regarded as a regulatory protein of hematopoietic cell biology for years. Nowadays increasing evidence supports that *ptpn6* plays a crucial role in the tumorigenesis and progression of some cancers. Nevertheless, most studies into its role in tumors to date have been limited to a specific type of cancer. Herein, the role of *ptpn6* for prognosis and immune regulation across 33 tumors was investigated, aiming to explore its function heterogeneity in pan-cancer and the clinical significance. The results showed that *ptpn6* can serve as a prognostic factor for a variety of cancers, and that it can play an important role in tumor immunity by affecting tumor infiltrating immune cells.

In this study, we not only have a landscape of the roles of *ptpn6* across different tumors, including the expression, prognostic, immune correlation, genetic alteration, epigenetic alterations, relevant cellular pathway and function, but also provide insight into presenting a potential prognosis biomarker and immunotherapy target. It also reflects the close relationship between *ptpn6* epigenetic modifications (such as methylation), *ptpn6* gene expression, cancer immune environment and prognosis. Moreover, interestingly, *ptpn6* can be served as an independent prognostic factor of many tumors and for different tumors, its expression level will bring different or even completely opposite prognostic outcomes. This suggest a complex correlation between *ptpn6* expression and the survival of different types of cancer. These findings aid in understanding the role of *ptpn6* in tumorigenesis and development, and have important implications for tumor diagnosis and immunotherapy in the era of precision medicine.

## Electronic supplementary material

Below is the link to the electronic supplementary material.

Supplementary Material 1

Supplementary Material 2

Supplementary Material 3

Supplementary Material 4

Supplementary Material 5

Supplementary Material 6

Supplementary Material 7

## Data Availability

All data generated or analyzed during this study are included in the specified repository (http://timer.cistrome.org/, http://gepia2.cancer-pku.cn/, https://kmplot.com/analysis/, https://cistrome.shinyapps.io/timer/, https://string-db.org/, https://david.ncifcrf.gov/, http://biocc.hrbmu.edu.cn/CancerSEA/ and http://www.cbioportal.org, http://ualcan.path.uab.edu/analysis.html).
