# Supplementary figures and images for: Pan-cancer analysis of the prognostic and immunological roles of SHP-1/ptpn6
Source: Sci Rep. 2024 Oct 4;14:23083. doi: 10.1038/s41598-024-74037-9 (PMC11452508; doi:10.1038/s41598-024-74037-9)

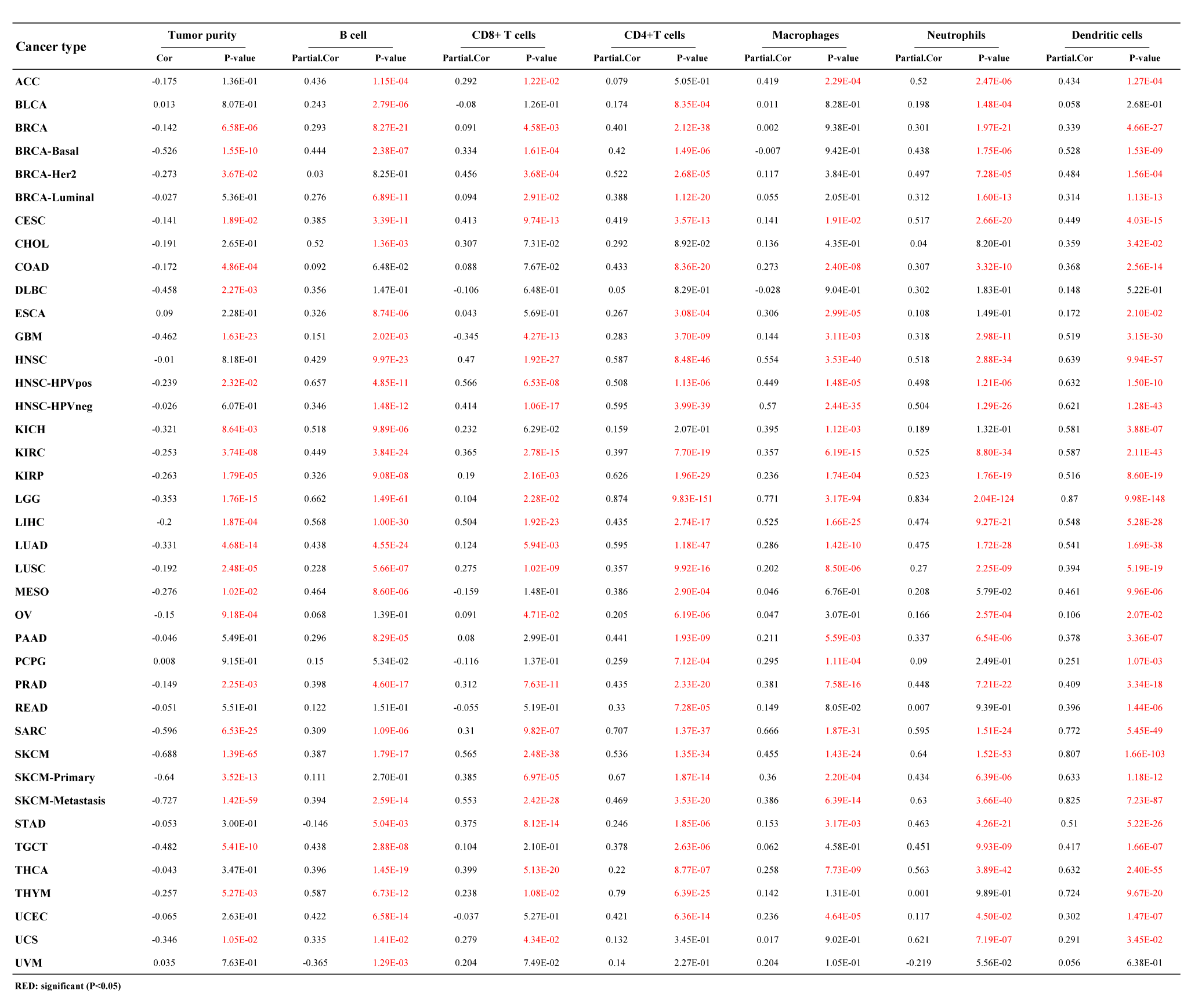

Supplement: Supplementary file 1 — Supplementary Material 1 [file 41598_2024_74037_MOESM1_ESM.tif]

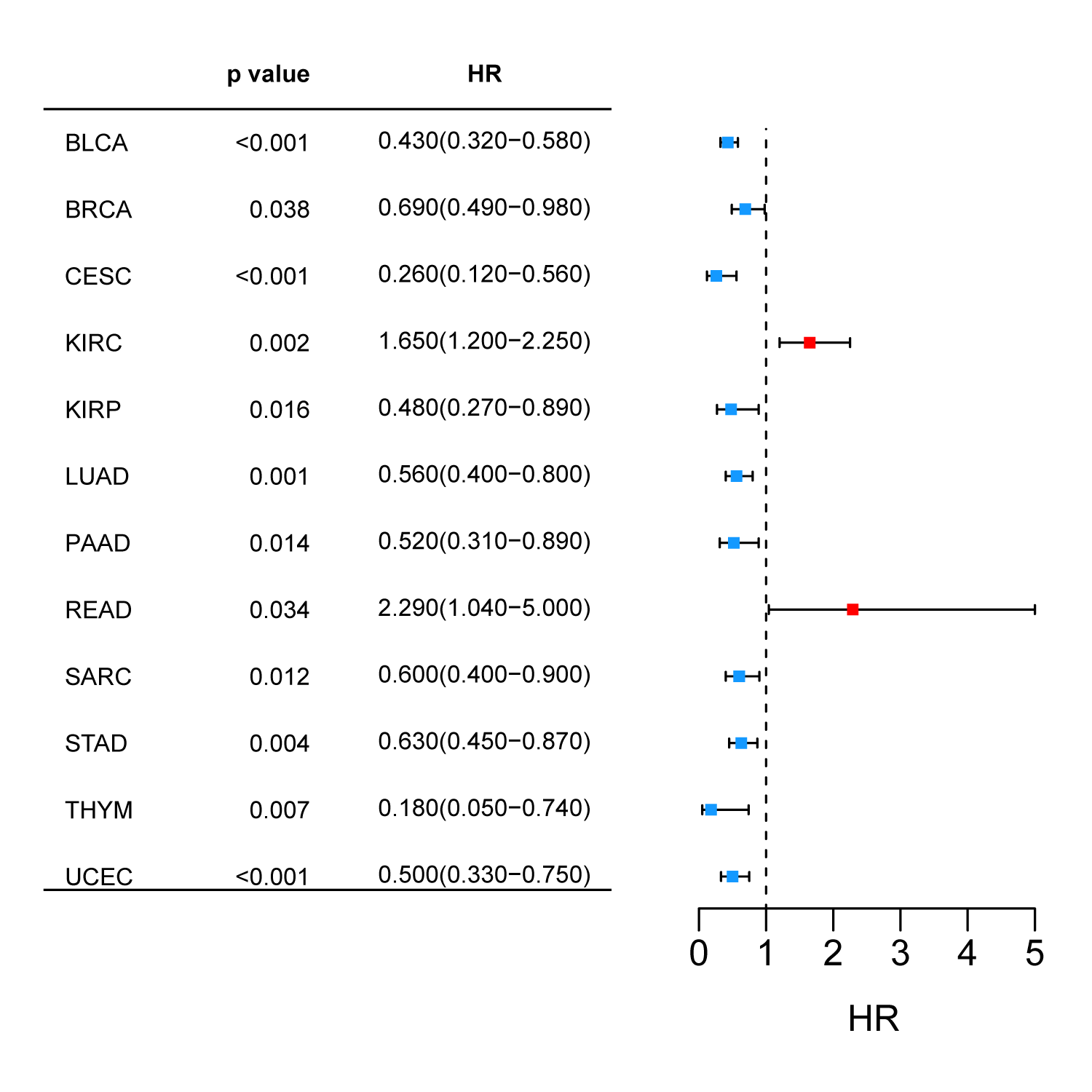

Supplement: Supplementary file 2 — Supplementary Material 2 [file 41598_2024_74037_MOESM2_ESM.tif]

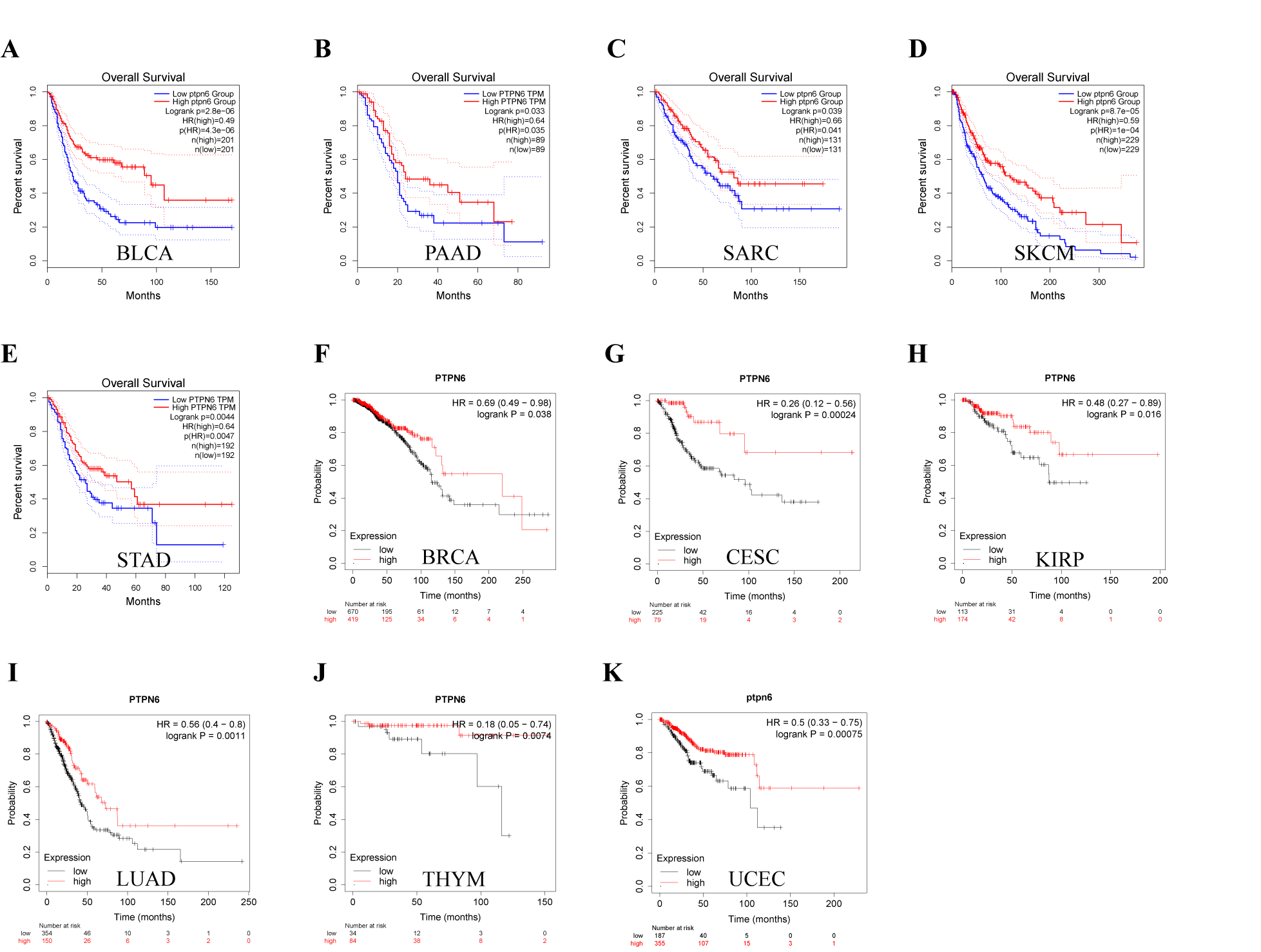

Supplement: Supplementary file 3 — Supplementary Material 3 [file 41598_2024_74037_MOESM3_ESM.tif]

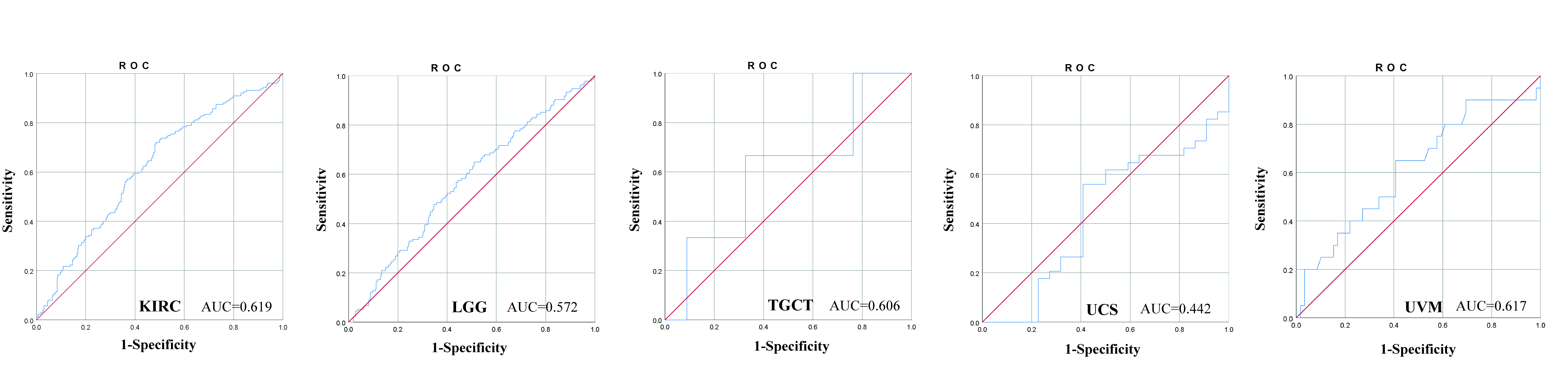

Supplement: Supplementary file 4 — Supplementary Material 4 [file 41598_2024_74037_MOESM4_ESM.tif]

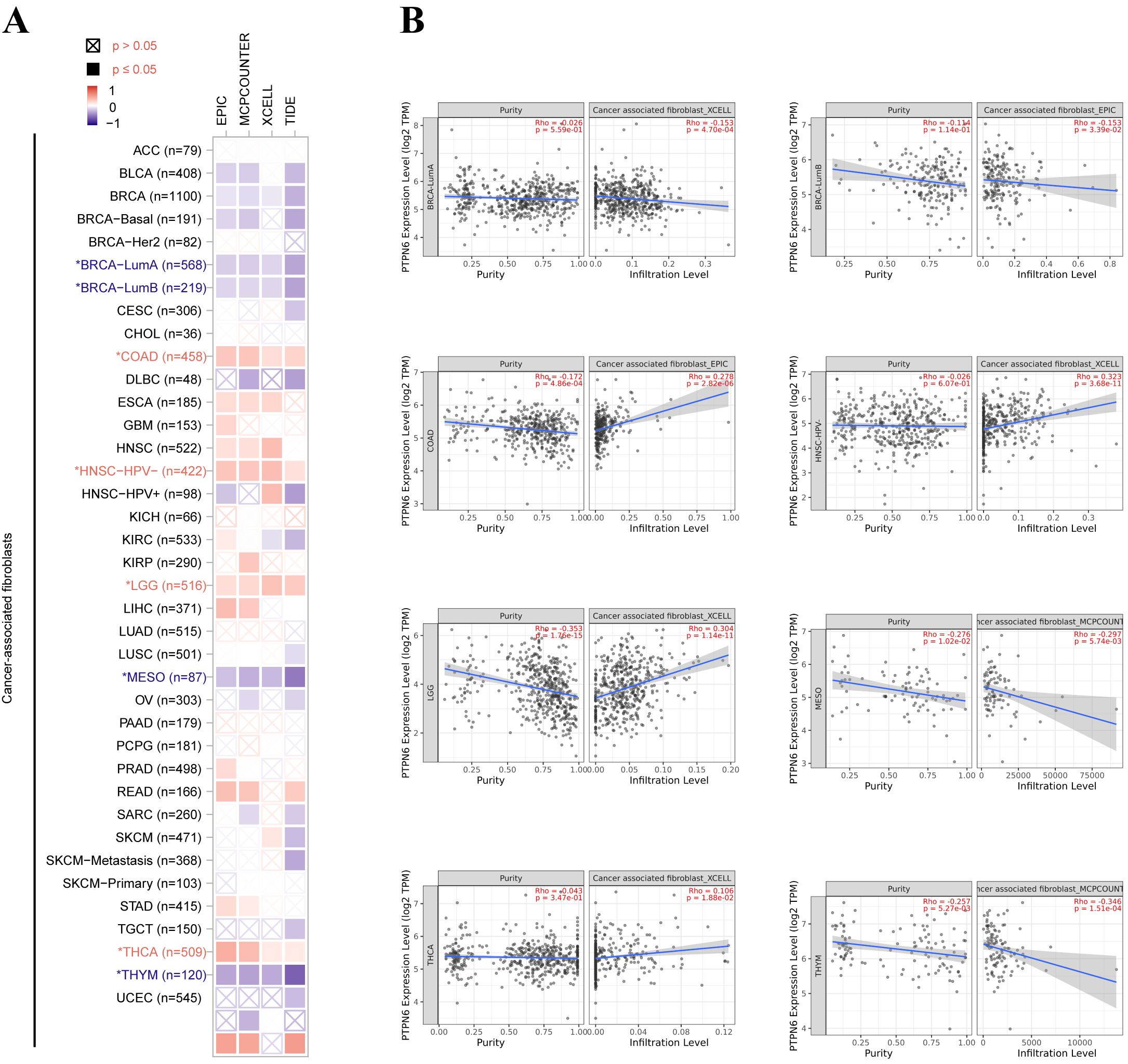

Supplement: Supplementary file 5 — Supplementary Material 5 [file 41598_2024_74037_MOESM5_ESM.tif]

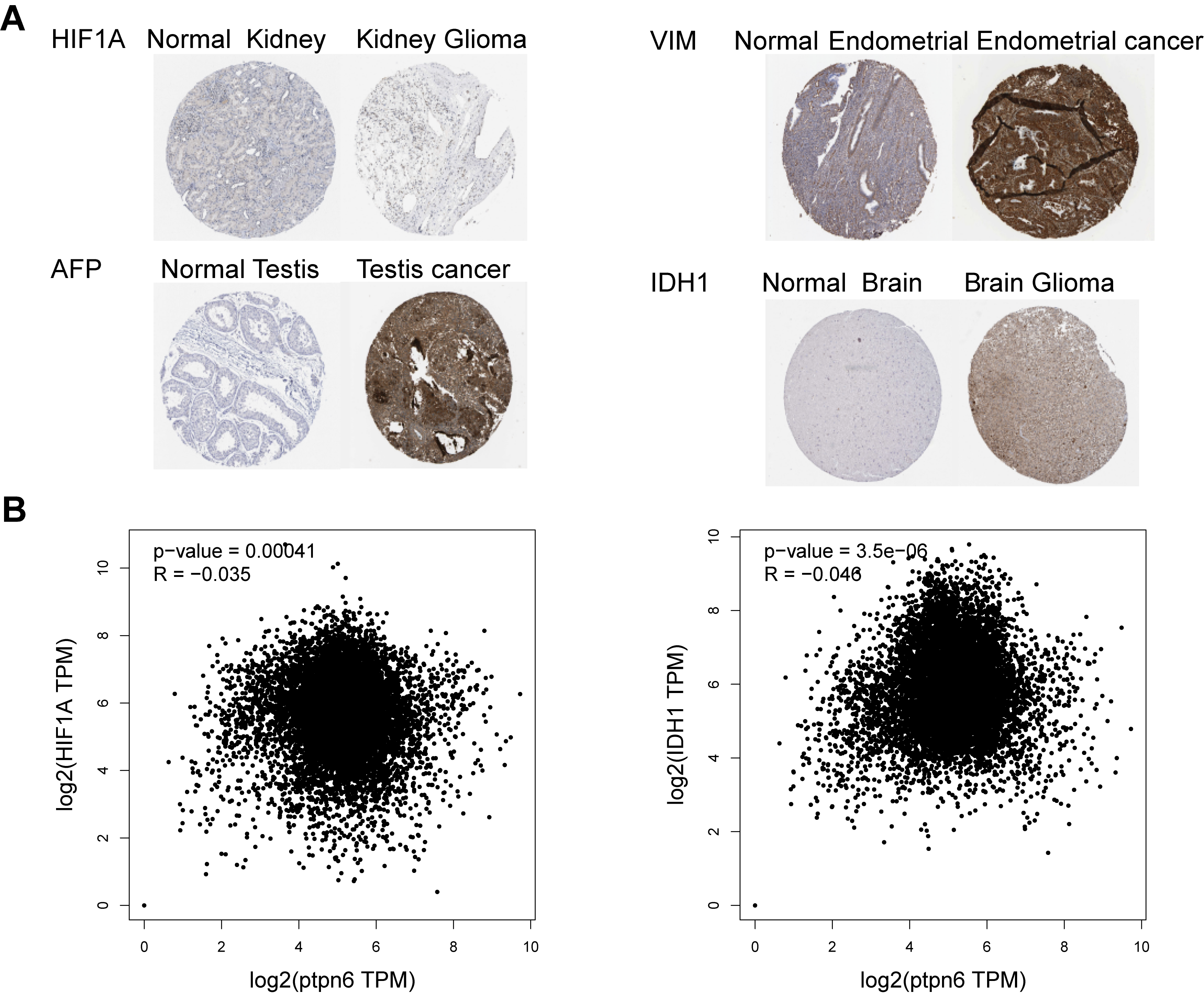

Supplement: Supplementary file 6 — Supplementary Material 6 [file 41598_2024_74037_MOESM6_ESM.tif]
