# Supplementary material for: Pan-cancer analysis of the prognostic and immunological roles of SHP-1/ptpn6
Source: Sci Rep. 2024 Oct 4;14:23083. doi: 10.1038/s41598-024-74037-9 (PMC11452508; doi:10.1038/s41598-024-74037-9)
Supplement: Supplementary file 7 — Supplementary Material 7 [file 41598_2024_74037_MOESM7_ESM.docx]

**Supplement file**

**Table S1** Correlation analysis of *ptpn6* expression and immune infiltration levels in 32 tumors.

**
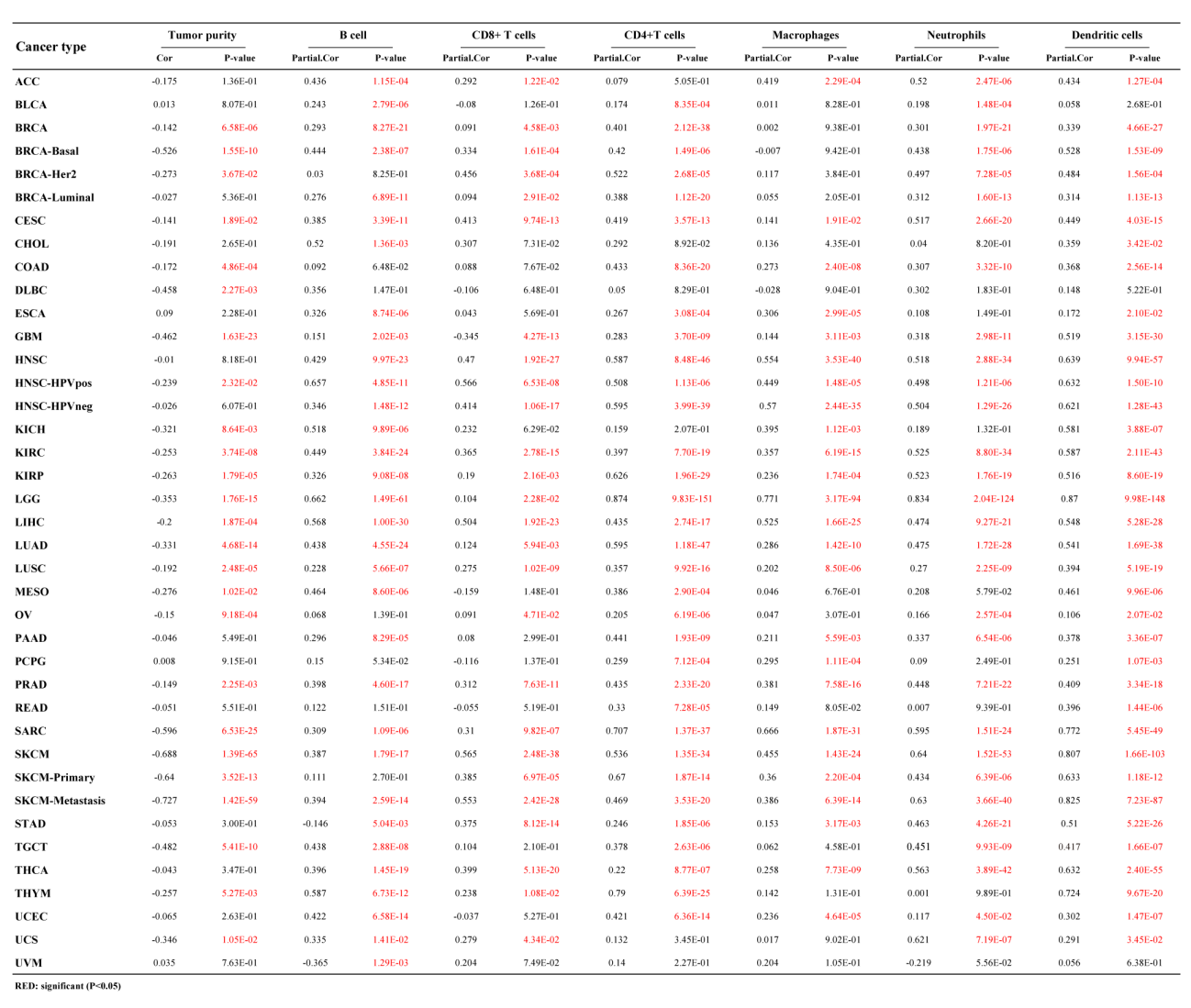
**

**
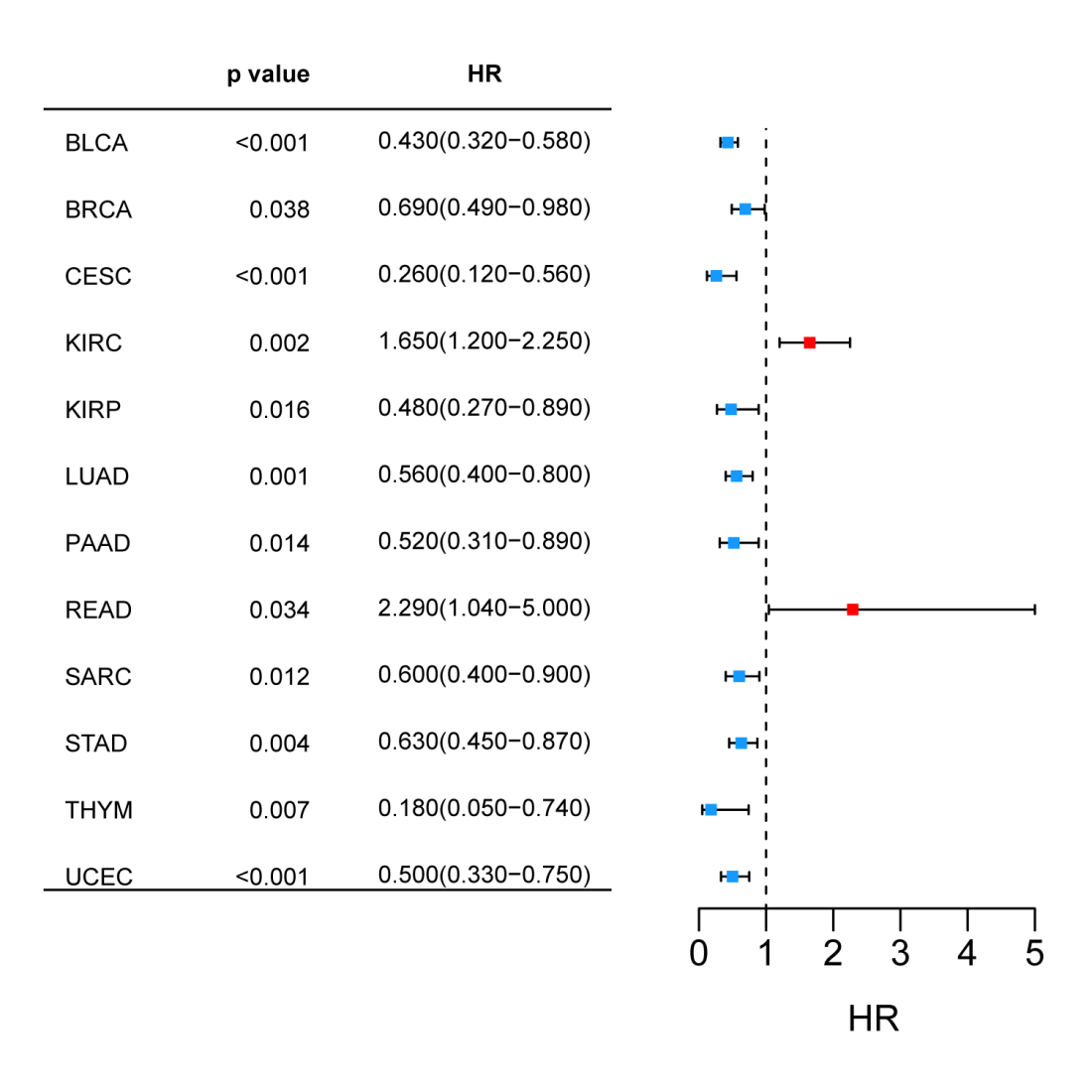
**

**Figure S1** The forest plot of the correlation between *ptpn6* expression and OS of 12 types of tumor (all *p*<0.05).

**
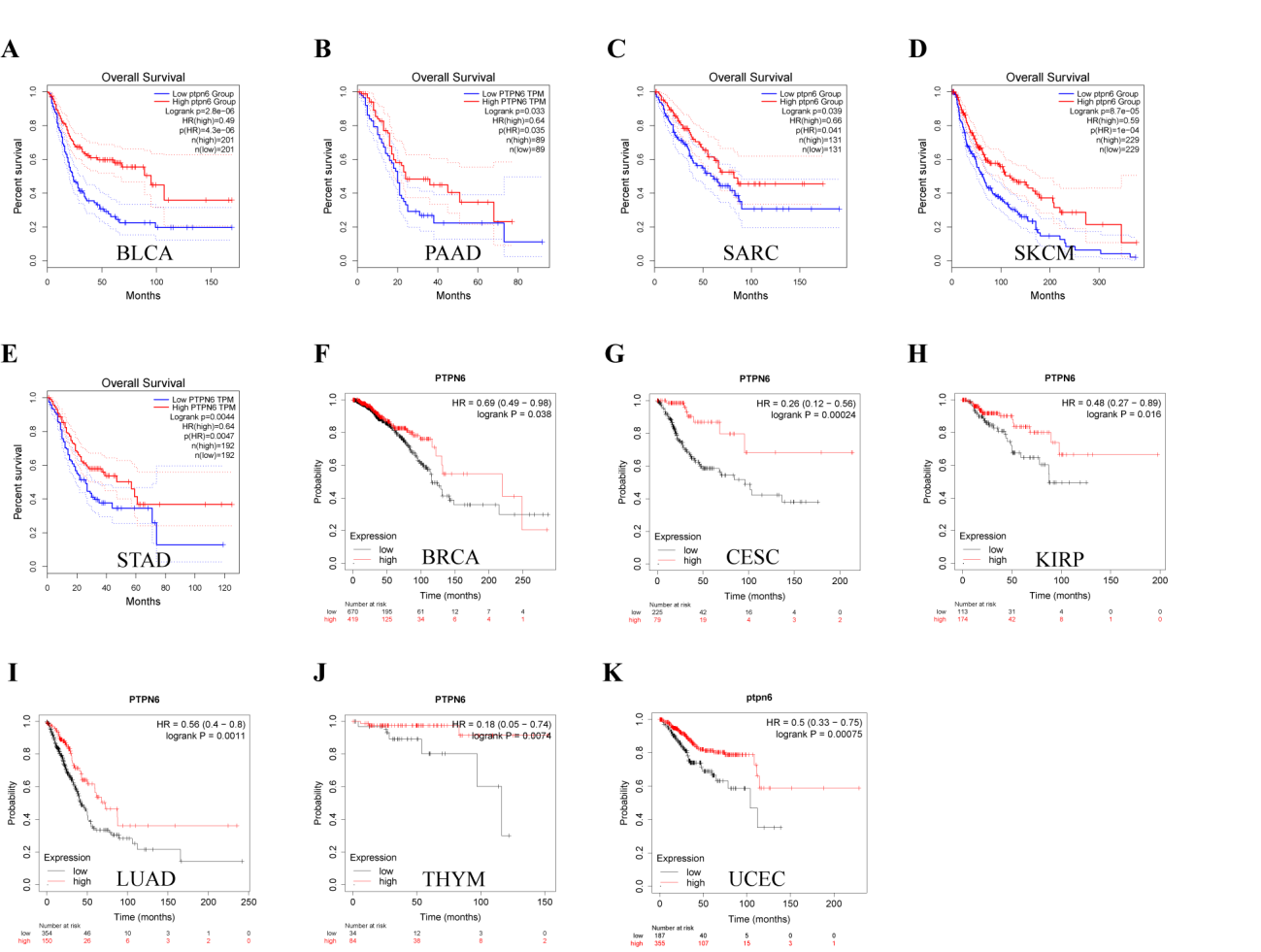
**

**Figure S2** Correlation analysis of *ptpn6* expression and OS in pan-cancer by Kaplan-Meier (A-E) and GEPIA2 (F-K) database.


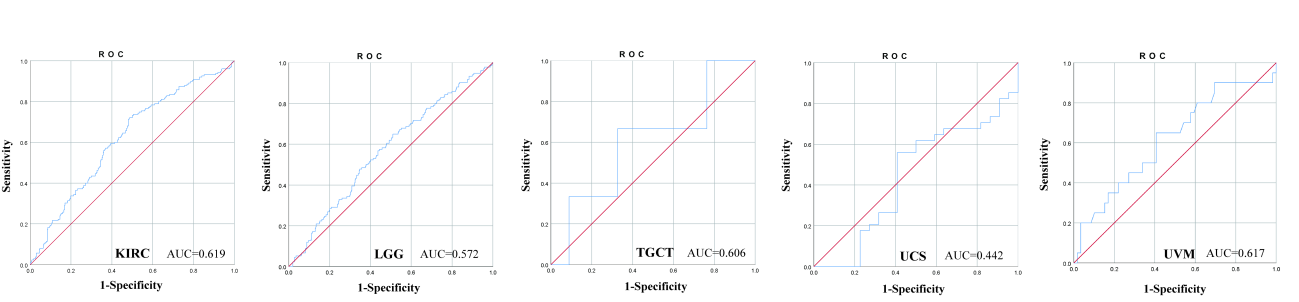


**Figure S3** The ROC curves of correlation between *ptpn6* expression and OS. The area under the curve (AUC) values of KIRC, LGG, TGCT, UCS and UVM (AUC =0.619, 0.572, 0.606, 0.442, 0.617, respectively) were computed.

**
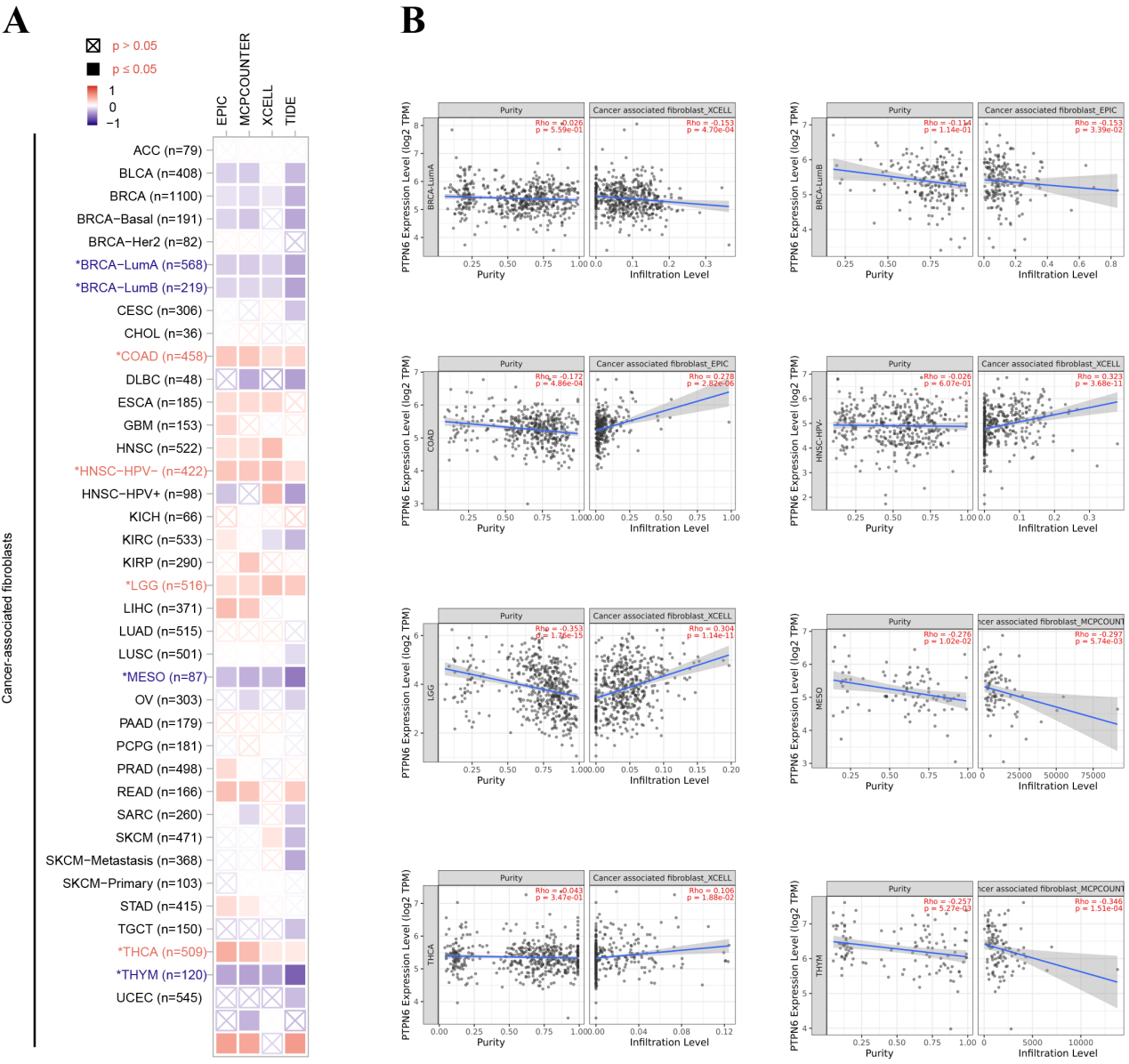
**

**Figure S4 Relationships between *ptpn6* and cancer-associated fibroblasts (CAFs) in the tumor microenvironment. (A)** The EPIC, MCPCOUNTER, XCELL and TIDE algorithms were used to correlate the CAF scores of different tumors with the expression of *ptpn6* gene. **(B)** The correlation of *ptpn6* expression and CAFs infiltration level in BRCA-LumA, BRCA-LumB, COAD, HNSC-HPV-, LGG, MESO, THCA, and THYM.

**Figure S5: Representative IHC images of key immune markers in various cancers and their normal tissues, and their correlation analysis with *ptpn6* expression. (A)** Representative IHC images showing the expression of HIF1A, AFP, IDH1 and VIM in kidney cancer, testicular cancer, brain glioma and uterine carcinoma, respectively. **(B)** The correlation of the expression between *ptpn6* and the immune markers HIF1A and IDH1 (R=-0.035, -0.046).
